# Genome‐Wide Analysis of DtxR and HrrA Regulons Reveals Novel Targets and a High Level of Interconnectivity Between Iron and Heme Regulatory Networks in *Corynebacterium glutamicum*

**DOI:** 10.1111/mmi.15376

**Published:** 2025-05-16

**Authors:** Aileen Krüger, Ulrike Weber, Julia Frunzke

**Affiliations:** ^1^ Forschungszentrum Jülich GmbH Institute for Bio‐ and Geosciences 1 Jülich Germany

**Keywords:** chromatin affinity purification and sequencing, heme, homeostasis, iron, transcription factors

## Abstract

Iron is vital for most organisms, serving as a cofactor in enzymes, regulatory proteins, and respiratory cytochromes. In *Corynebacterium glutamicum* , iron and heme homeostasis are tightly interconnected and controlled by the global regulators DtxR and HrrA. While DtxR senses intracellular Fe^2+^, HrrSA is activated by heme. This study provides the first genome‐wide analysis of DtxR and HrrA binding dynamics under varying iron and heme conditions using chromatin affinity purification and sequencing (ChAP‐Seq). We revealed 25 novel DtxR targets and 210 previously unrecognized HrrA targets. Among these, *metH,* encoding homocysteine methyltransferase, and *xerC,* encoding a tyrosine recombinase, were bound by DtxR exclusively under heme conditions, underscoring condition‐dependent variation. Activation of *metH* by DtxR links iron metabolism to methionine synthesis, potentially relevant for the mitigation of oxidative stress. Beyond novel targets, 16 shared targets between DtxR and HrrA, some with overlapping operator sequences, highlight their interconnected regulons. Strikingly, we demonstrate the significance of weak ChAP‐Seq peaks that are often disregarded in global approaches, but feature an impact of the regulator on differential gene expression. These findings emphasize the importance of genome‐wide profiling under different conditions to uncover novel targets and shed light on the complexity and dynamic nature of bacterial regulatory networks.

## Graphical abstract

Iron is essential for life, yet excessive levels can quickly lead to cellular damage via oxidative stress, underscoring the need for global regulators of iron and heme homeostasis. In this study, we present a genome‐wide analysis of the binding patterns of DtxR and HrrA in *Corynebacterium glutamicum* . Our findings highlight the interconnected nature of DtxR and HrrA networks and underscore the value of condition‐specific analyses for a deeper understanding of bacterial adaptation to environmental changes.

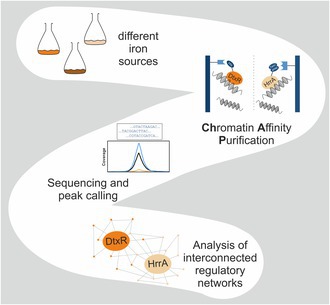

## 1 Introduction

Iron is a crucial trace element required by nearly all organisms due to its indispensable role as a cofactor for vital enzymes and regulatory proteins, as well as its integral presence in cytochromes involved in the respiratory chain (Andrews et al. 2003; Cornelis et al. 2011). Despite its biological significance, iron poses a unique challenge as it exhibits very low solubility under aerobic conditions, making it a scarce nutrient in many environments. To overcome this limitation, organisms have evolved intricate measures ensuring sufficient iron supply and storage (Andrews et al. 2003). However, while ferrous iron (Fe^2+^) is essential for numerous cellular processes, elevated levels can be toxic to cells. This toxicity primarily arises from its reaction with hydrogen peroxide (H_2_O_2_) via the Fenton reaction, which generates highly reactive oxygen species (ROS) (Cornelis et al. 2011). Consequently, iron homeostasis is critical for cellular fitness and is maintained by sophisticated regulatory networks integrating various parameters, such as the availability of iron, oxidative stress levels, or access to alternative iron sources (e.g., heme). Notably, for the latter, the homeostasis of iron and heme is intricately interconnected in almost all organisms, which is also reflected on the level of regulatory networks.

The actinomycetotal organism *Corynebacterium glutamicum* represents a model organism for studying regulatory mechanisms underlying iron homeostasis. In this species, iron homeostasis is governed by the global iron‐dependent regulator DtxR and through the activity of two paralogous, heme‐responsive two‐component systems (TCS), HrrSA and ChrSA (Frunzke et al. 2011; Heyer et al. 2012). While DtxR senses intracellular Fe^2+^ levels through direct binding of ferrous ions (Schmitt and Holmes 1993), the histidine kinases HrrS and ChrS respond to heme via an intramembrane sensing mechanism (Keppel et al. 2018). The DtxR protein was first discovered as “*d*iphtheria *t*o*x*in *r*epressor” in the related pathogenic species *Corynebacterium diphtheriae* where it controls expression of the toxin in an iron‐dependent manner (Boyd et al. 1990; Pappenheimer and Johnson 1936). Meanwhile, DtxR‐like proteins were shown to be conserved in many Gram‐positive organisms and well‐characterized examples include IdeR in *Mycobacterium tuberculosis* (Gold et al. 2001) or MntR in *Staphylococcus aureus* (Ando et al. 2003). In Corynebacteria, DtxR was shown to be involved in the regulation of more than 60 genes related to iron acquisition, storage as well as iron–sulfur cluster assembly (Brune et al. 2006; Wennerhold and Bott 2006). DtxR is active as a dimer in complex with Fe^2+^ in iron sufficient conditions. Under iron limiting conditions, Fe^2+^ dissociates from DtxR rendering the protein inactive (White et al. 1998). Notably, DtxR also integrates into broader regulatory networks by acting as a repressor of genes encoding the key transcriptional regulators *ripA* (*r*epressor of *i*ron *p*roteins) and *hrrA* (*h*eme‐*r*esponsive response *r*egulator), a component of the HrrSA TCS. Besides *hrrA*, DtxR controls further genes involved in heme homeostasis, including the heme oxygenase (*hmuO*) or the heme uptake system (*hmuTUV*) (Drazek et al. 2000; Wennerhold and Bott 2006) indicating that there is considerable overlap of the iron‐ and heme‐responsive regulons governed by DtxR and HrrA in Corynebacteria.

The regulation of heme homeostasis via TCS is a widely conserved strategy in Gram‐positive bacteria (Krüger et al. 2022). In *C. glutamicum* , the two paralogous TCSs HrrSA and ChrSA are dedicated to sensing and responding to heme (Frunzke et al. 2011; Heyer et al. 2012). ChrSA is crucial for heme detoxification specifically by activating the expression of *hrtBA* encoding a heme exporter (Hentschel et al. 2014; Heyer et al. 2012; Keppel et al. 2019). In contrast, HrrSA represents a global regulatory system orchestrating the expression of more than 200 target genes involved in heme biosynthesis, respiration, and cell envelope remodeling (Keppel et al. 2020).

Several previous studies focused on the elucidation of the DtxR regulon or on the control of single components, providing valuable insights into its role in iron homeostasis (Boyd et al. 1990; Brune et al. 2006; Kunkle and Schmitt 2003, 2005; Wennerhold and Bott 2006). However, despite this progress, so far no study has focused on the in vivo dynamics and genome‐wide binding patterns of this global regulator and its potential interference with other networks. In this context, genome‐wide approaches hold tremendous potential to fill these gaps by revealing the binding dynamics, structural roles, and regulatory influence of DtxR and HrrA on a global scale.

Within this study, we followed an integrative approach using *ch*romatin *a*ffinity *p*urification and *seq*uencing (ChAP‐Seq) aiming to investigate the binding dynamics of the two global iron‐ and heme‐responsive regulators DtxR and HrrA, respectively. We show significant condition‐specific effects on the global binding pattern leading to the identification of several novel targets, thereby connecting DtxR and HrrA to broader cellular processes including DNA topology and repair as well as oxidative stress response. Additionally, our study highlights the importance of weak—often overlooked—binding sites, which may have prominent effects on transcriptional outcomes. Consequently, our genome‐wide studies provided systems‐level insights into the binding dynamics, interaction, and interference of the two global regulators orchestrating iron‐ and heme homeostasis in *C. glutamicum* .

## 2 Experimental Procedures

### 2.1 Bacterial Strains and Growth Conditions

The bacterial strains used within this study are listed in Table S1. *Escherichia coli* strains for cloning purposes were cultivated in Lysogeny Broth (Difco LB, Heidelberg, Germany) media, shaking at 37°C. If appropriate, 50 μg mL^−1^ kanamycin was added to the media for selection.

For a first pre‐culture, the *Corynebacterium glutamicum* ATCC 13032 wild type strain or its derivatives were cultivated in liquid BHI (brain heart infusion, Difco BHI, BD, Heidelberg, Germany), inoculated from a fresh agar plate (12 mL in 100 mL baffled shaking flask for ChAP‐Seq cultivation, 1 mL in deep‐well plates for microtiter cultivation). Incubation followed shaking at 30°C for approximately 8 h. A second pre‐culture was performed using the minimal medium CGXII supplemented with 2% (w/v) glucose (1 g L^−1^ K_2_HPO_4_, 1 g L^−1^ KH_2_PO_4_, 5 g L^−1^ urea, 42 g L^−1^ MOPS, 13.25 mg L^−1^ CaCl_2_ · 2 H_2_O, 0.25 g L^−1^ MgSO_4_ · 7 H_2_O, 10 mg L^−1^ FeSO_4_ · 7 H_2_O, 10 mg L^−1^ MnSO_4_ · H_2_O, 0.02 mg L^−1^ NiCl_2_ · 6 H_2_O, 0.313 mg L^−1^ CuSO_4_ · 5 H_2_O, 1 mg L^−1^ ZnSO_4_ · 7 H_2_O, 0.2 mg L^−1^ biotin, 30 mg L^−1^ 3,4‐dihydroxybenzoate (PCA), 20 g L^−1^ D‐glucose, pH 7.0) (Keilhauer et al. 1993). For ChAP‐Seq experiments, 12 mL pre‐culture 1 were transferred to 200 mL medium in a 1 L baffled shaking flask, while for the microtiter cultivation, 100 μL were used in 900 μL in a deep‐well plate. For the heme condition, no iron was added to this pre‐culture to starve these cells from iron, while for the iron excess condition, the standard amount of iron was used (36 μM FeSO_4_). For the iron depletion condition, a reduced amount of 1 μM FeSO_4_ was added to the pre‐culture. Incubation was performed at 30°C shaking for approximately 16 h. From this overnight culture, the main culture was inoculated to an OD_600_ of 3 in CGXII with 2% glucose and either 100 μM FeSO_4_ (iron excess condition) or 0 μM FeSO_4_ but 4 μM hemin (throughout this paper further referred to as heme) (heme condition) or 0 μM FeSO_4_ (iron depletion condition) shaking at 30°C. For ChAP‐Seq, this was cultivated in 1 L in 5 L baffled shaking flasks. For microtiter cultivation in 48‐well microtiter FlowerPlates in a BioLector I microbioreactor cultivation system (Beckman Coulter GmbH, Aachen, Germany) (Kensy et al. 2009) for online‐monitoring. FlowerPlates were sealed with a gas‐permeable sealing foil (VWR, Radnor, United States). In the microbioreactor cultivation system, backscatter (a.u.) was measured in 30 min intervals as scattered light at *λ*: 620 nm (signal gain: 20), while venus‐fluorescence was measured at *λ*ex: 508 nm /*λ*em: 532 nm (signal gain: 80). Specific fluorescence (a.u.) was calculated by dividing the venus‐signal by the backscatter signal for each measurement. For cultivation with plasmids, 25 μg mL^−1^ kanamycin was added.

### 2.2 Recombinant DNA Work and Cloning Techniques

Standard molecular methods were performed according to standard protocols (Sambrook and Russell 2001). DNA fragments were amplified via polymerase chain reactions (PCR), using chromosomal DNA of *C. glutamicum* ATCC 13032 as the template and the oligonucleotides listed in Table S2. Preparation was performed as described previously (Eikmanns et al. 1994).

Plasmids were constructed by enzymatically assembling the generated DNA fragments into a cut vector backbone using Gibson assembly (Gibson et al. 2009). Sequencing of the final plasmid was performed by Eurofins Genomics (Ebersberg, Germany).

For the integration of the His‐Tag and a linker (GGGS_2_) at the C‐terminus of DtxR in the genome of *C. glutamicum* , the suicide vector pK19‐*mobsacB* was used (Schäfer et al. 1994). Electrocompetent *C. glutamicum* cells were transformed with the isolated plasmid by electroporation (van der Rest et al. 1999). Then, the first and second recombination events were performed and verified as described in previous studies (Niebisch and Bott 2001). The respective deletion was reviewed by amplification and sequencing (Eurofins Genomics, Ebersberg, Germany).

### 2.3 Reverse Transcription Polymerase Chain Reaction (RT‐qPCR)

For analysis of the *C. glutamicum* strain with a tagged DtxR variant (::*dtxR*‐C‐linker‐His), cultivation as described in 2.1 in deep‐well plates was performed, harvesting cells in ice‐falcons at an OD_600_ of 5 in exponential phase. Using the Luna One‐Step RT‐qPCR Kit (New England BioLabs, Frankfurt am Main) according to the manufacturer's instructions, qPCR was performed in the qTower (Analytik Jena, Jena). As a reference gene for normalization, the housekeeping gene *ddh* was used (Frunzke et al. 2008), besides the target gene *dtxR*. Analysis followed using qPCRsoft 3.1 (Analytik Jena, Jena) and fold change was calculated according to the 2^−ΔΔCt^ method (Livak and Schmittgen 2001).

### 2.4 Chromatin Affinity Purification‐Sequencing (ChAP‐Seq)—Sample Preparation

The protocol for obtaining DNA was adapted to recent studies (Keppel et al. 2020; Pfeifer et al. 2016). The strains *C. glutamicum* ATCC 13032::*dtxR*‐C‐linker‐His and *C. glutamicum* ATCC 13032::*hrrA*‐C‐twin‐Strep were cultivated as described in 3.1. After 2 h cultivation at 30°C in a rotary shaker, cells were harvested (5000 x *g*, 4°C, 10 min). The cell pellets were washed once with CGXII without MOPS and then the cells were incubated in 20 mL CGXII without MOPS and 1% formaldehyde for 20 min at RT to cross‐link the regulator protein to the DNA. The reaction was stopped via incubation with 125 mM glycine for 5 min. Then, cells were washed three times with either TNI20 (20 mM Tris–HCl, 300 mM NaCl, 20 mM Imidazol, pH 8) or buffer A (100 mM Tris–HCl, pH 8; importantly w/o EDTA, to avoid the chelation of iron ions required for DtxR activity) and the pellets were stored overnight at −80°C. For cell disruption and purification, the pellets were resuspended in approximately 20 mL TNI20 or buffer A with cOmplete protease inhibitor (Roche, Germany) and 2 mg RNase A (AppliChem, Darmstadt, Germany) and disrupted at 40,000 psi using the Multi Shot Cell Disrupter (I&L Biosystems, Germany). To shear the chromosomal DNA, the samples were sonified 2 × 20 s with the Branson Sonifier 250 (Branson Ultrasonics Corporation, CT, USA) and finally, the supernatant was collected after ultra‐centrifugation (150,000 x *g*, 4°C, 1 h).

The DNA, which was bound by the His‐tagged DtxR, was purified using Ni‐NTA Agarose column material (Thermo Fisher Scientific, USA) according to the manufacturer's instructions to the gravity flow protocol. Washing of the column was performed using TNI20, and the tagged protein and the bound DNA were eluted with TN buffer with rising imidazole concentrations (20 mM Tris–HCl, 300 mM NaCl, 50/100/200/400 mM Imidazol, pH 8) in each 1 mL. A Bradford assay was performed to evaluate which of the eluted fractions would be pooled for further DNA preparation. In the end, these were the last three of TNI50 elution and the first three of TNI100.

The DNA bound by the twin‐Strep‐tagged HrrA was purified using Strep‐Tactin XT Superflow column material (IBA Lifesciences, Germany) according to the manufacturer's instructions for the gravity flow protocol. Washing of the column was performed using buffer W (100 mM Tris–HCl, 150 mM NaCl, pH 8) and the tagged protein together with the bound DNA was eluted with buffer E (100 mM Tris–HCl, 150 mM NaCl, 15 mM D‐biotin, pH 8).

After the purification, 1% (w/v) SDS was added to the eluted (and pooled) fractions and incubated overnight at 65°C. Digestion of the protein was accomplished with the addition of 400 μg mL^−1^ Proteinase K (AppliChem GmbH, Germany) at 55°C for 2 h. Purification of DNA followed by adding Roti‐Phenol/Chloroform/Isoamylalcohol (Carl Roth GmbH, Germany) in a 1:1 ratio to the samples and consequent separation of the organic phase using Phase Lock Gel (PLG) tubes (VWR International GmbH, Germany) according to the manufacturer's instructions. The aqueous phase was removed and combined with 0.1 volume of 3 M sodium acetate and twice the volume of ice‐cold ethanol. The mixture was then incubated at −20°C for 2 h. This was followed by centrifugation at 16,000 x *g* for 10 min at 4°C. The DNA was precipitated by adding ice‐cold 70%(v/v) EtOH. Following an additional centrifugation, the supernatant was carefully removed. The DNA was then dried for at least 3 h at 50°C and subsequently eluted in dH_2_O.

### 2.5 ChAP‐Seq—Sequencing and Peak Analysis

The isolated DNA fragments (Section 2.4) were used for library preparation and indexing with the TruSeq DNA PCR‐free sample preparation kit (Illumina, Chesterford, UK) according to the manufacturer's instructions, leaving out the DNA size selection steps. Libraries were quantified using the KAPA library quant kit (Peqlab, Bonn, Germany) and normalized for pooling. These pooled libraries were sequenced using the MiSeq device (Illumina) (paired‐end sequencing, read length: 2 × 150 bases). Data analysis as well as base calling was performed with the Illumina instrument software, yielding fastq output files. Further data analysis was based and modified according to Keppel et al. (2020). To remove PCR amplification artifacts, sequencing data were collapsed for each sample. The processed fastq files were mapped to accession NC_003450.3 as the *C. glutamicum* reference genome. This was done using Bowtie2 with the following parameters: –ignore‐quals –local –very‐sensitive‐local –rfg 9,5 –rdg 9,5 –score‐min L,40,1.2 ‐k 8 –no‐unal –no‐mixed –threads 8 ‐I 40 ‐X 800 (Langmead and Salzberg 2012; Langmead et al. 2019). The genomic coverage was convoluted with a second order Gaussian kernel. The kernel was truncated at 4 sigmas and expanded to the expected peak width. The expected peak width was predicted using the following procedure: (i) All peaks higher than 3 mean coverage were detected. (ii) Points at which coverage dropped below half of the maximal peak height were detected and the distance between those was set as peak width. (iii) The estimated peak width was fixed equal to the median peak width. Convolution profiles were scanned to allow identification of the regions where the first derivative changes from positive to negative. Each of these regions was determined as a potential peak with an assigned convolution score (convolution with second order Gaussian kernel centered at the peak position). Filtered peaks were normalized for inter‐sample comparisons and the sum of coverages of all detected peaks was negated from the total genomic coverage. This difference was used as a normalization coefficient and was divided by peak intensities.

## 3 Results

### 3.1 Genome‐Wide Profiling of DtxR and HrrA DNA‐Binding Under Different Iron‐Conditions

To gain an integrated view on the global iron‐ and heme regulatory networks controlled by DtxR and HrrA, we performed ChAP‐Seq experiments revealing their genome‐wide binding patterns under different iron conditions. For this purpose, tagged variants of both proteins were constructed to enable affinity purification. Fusion with a twin‐Strep‐tag was already successfully implemented for HrrA (Keppel et al. 2020). To ensure that tagging does not interfere with DtxR's function, it was necessary to include a flexible linker sequence between DtxR and the His‐tag (Chen et al. 2013) (Figure S1A). Growth analysis of the strain expressing the C‐terminal His‐tagged DtxR variant including the linker showed that there was no growth disturbance neither under standard conditions nor under iron excess conditions, while an N‐tagged variant featured a significant growth defect (Figure S1B). Notably, also qPCR analysis confirmed that the C‐terminal His‐tagged version of *dtxR* is expressed at wild‐type levels in the presence of iron (2^−ΔΔCt^_iron excess_ = 1.11 ± 0.05; 2^−ΔΔCt^_standard_ = 1.44 ± 0.15, Figure S1C).

**FIGURE 1:**
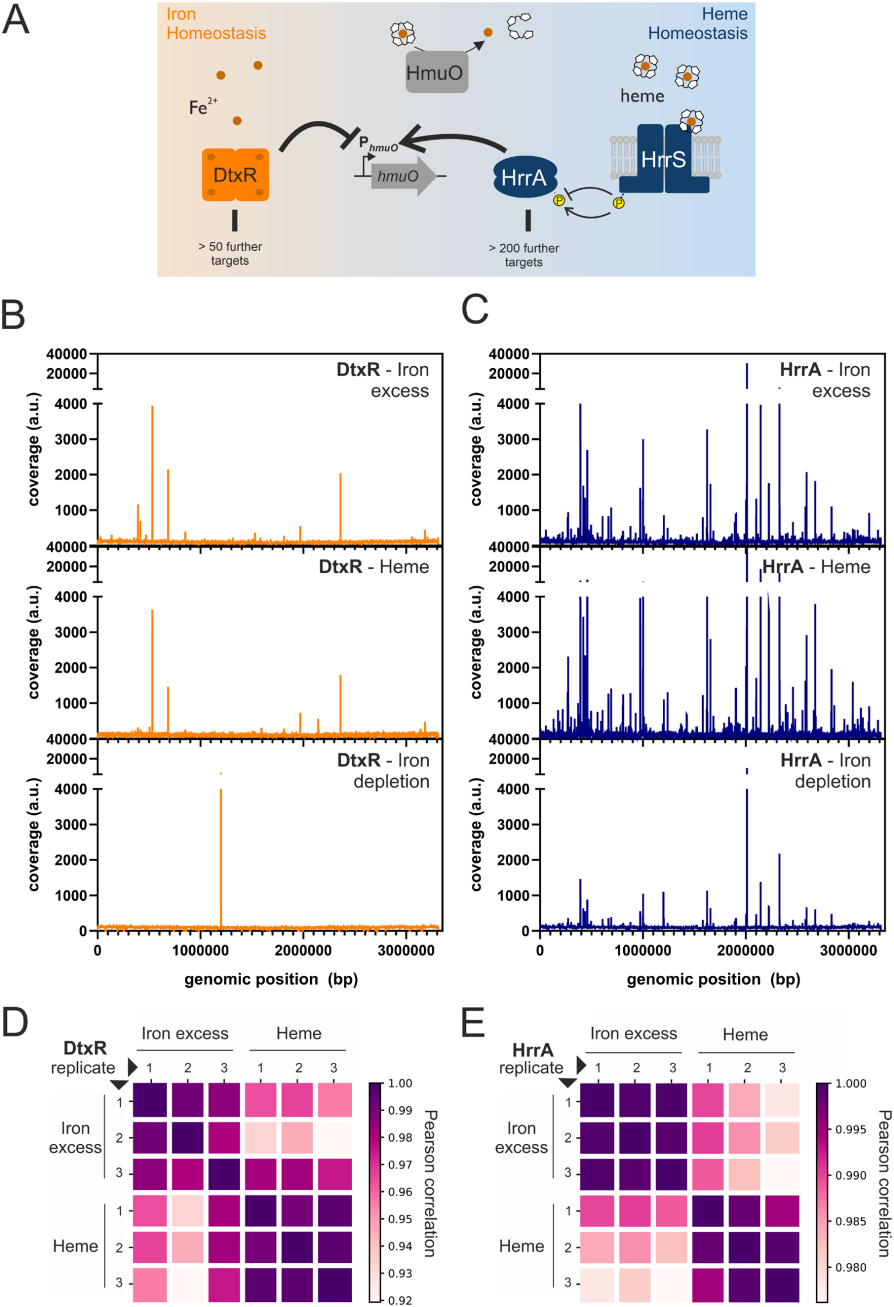
Genome‐wide profiling of DtxR and HrrA DNA‐binding in *Corynebacterium glutamicum* . (A) Schematic overview of the global iron and heme regulators DtxR and HrrA and the shared target gene *hmuO* encoding for a heme oxygenase. In (B) and (C), mapping of ChAP‐seq reads for DtxR (orange) and HrrA (blue), respectively, to the *C. glutamicum* ATCC 13032 genome is shown. DNA was obtained by affinity purification of DtxR and HrrA from cultures grown under iron excess (top, 100 μM FeSO_4_), heme (middle, 4 μM heme) and iron depletion (bottom, 0 μM FeSO_4_). Shown is one representative of each triplicate. Further replicates are shown in Figure S3. Note that the outstanding peak in the iron depletion condition for DtxR, found also smaller in HrrA binding, is a cryptic one and not real, resulting from technical issues as depicted in Figure S4. (D) and (E) represent the Pearson correlation of identified peaks among all replicates for DtxR and HrrA respectively binding at the two different conditions of iron excess (100 μM FeSO_4_) and heme (4 μM).

The genome‐wide binding patterns of the two regulators DtxR and HrrA were analyzed under iron excess conditions (100 μM FeSO_4_), with heme as an alternative iron source (4 μM heme) and under iron‐depleted conditions (0 μM FeSO_4_) (Figure 1A). The general procedure is shown schematically in Figure S2. In direct comparison, it could be confirmed that HrrA binds to a higher number of genomic targets in comparison to DtxR. Overall, a very similar binding pattern was observed for growth on iron excess and heme, respectively. This is not surprising and reflects efficient iron and heme homeostatic processes stabilizing the intracellular pool of chelatable Fe^2+^ and heme. Under iron excess conditions, sufficient heme synthesis is supported (Frunzke et al. 2011; Layer 2021), while in iron‐starved but heme‐rich conditions, heme serves as an alternative iron source via heme oxygenase HmuO (Wilks and Schmitt 1998). The iron depletion condition reflects the significantly lower activity of these regulators with less iron: While HrrA still binds to several of its high‐affinity targets, binding of DtxR is almost completely abolished, ultimately leading to the strong upregulation of the iron starvation response. Pearson correlation confirmed strong consistency within triplicates of the same samples, while revealing significant differences between samples obtained from iron‐ or heme‐grown cells. This highlights subtle but significant differences in genome‐wide binding patterns observed under iron and heme conditions (Figure 1B,C). Notably, samples obtained from cells with a native DtxR protein (without tag) grown under iron excess conditions served as a negative control. Here, no unspecific binding was observed (Figure S3).

#### 3.1.1 Condition‐Specific Analysis Revealed Several New DtxR Targets

**FIGURE 2:**
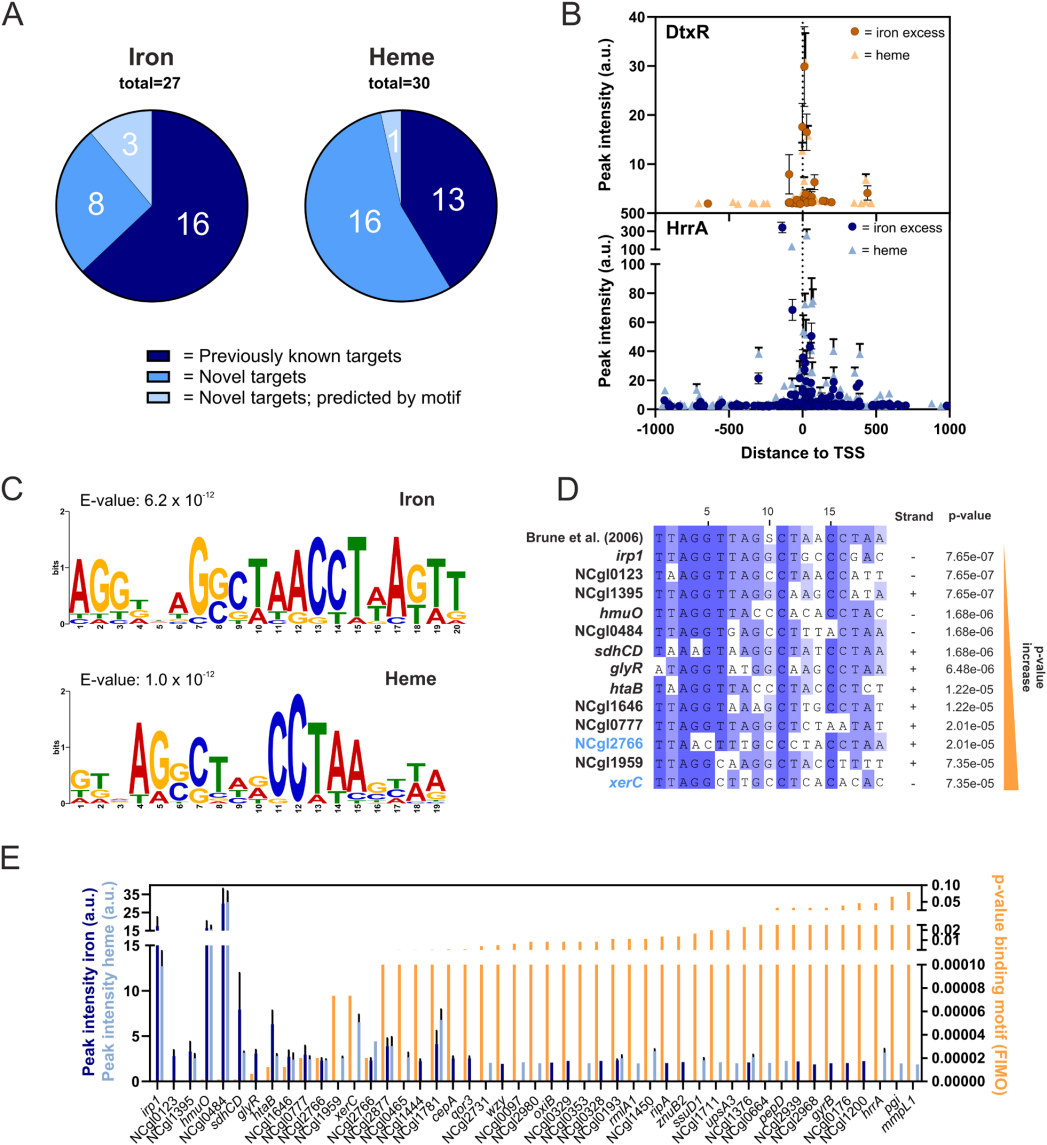
Global analysis of DtxR peaks showed correlation between peak intensity and motif conservation. (A) Pie charts comparing the number of targets bound by DtxR in this ChAP‐Seq experiment that were already confirmed by previous studies (Brune et al. 2006; Wennerhold and Bott 2006) (previously known targets), novel targets, and those that were previously predicted from motif search, but could not be found in vitro (novel targets; predicted by motif). (B) The ChAP‐Seq peak intensities in arbitrary units for the iron excess (darker color) or heme (lighter color) condition were correlated to the peak distance relative to the transcriptional start site (TSS) for DtxR (orange) and HrrA (blue), respectively. (C) MEME‐ChIP motif prediction of the DtxR binding motif based on ChAP‐Seq binding peaks extracted from iron excess and heme conditions (Bailey and Elkan 1994). (D) MUSCLE (Multiple Sequence Comparison by Log‐Expectation) alignment (Madeira et al. 2024) of previously predicted DtxR binding motif (Brune et al. 2006) and all fitting motifs found throughout the ChAP‐Seq targets using FIMO (*p* < 1.0e^−05^) (Grant et al. 2011), sorted from lowest to highest *p*‐value. Results with a higher *p*‐value can be found in Figure S7. Targets in light blue represent novel, so far unknown targets. (E) Correlation of peak intensities in iron excess (dark blue) or heme (light blue) condition with the *p*‐value of the binding motif as calculated by FIMO (Grant et al. 2011).

For the iron‐responsive regulator DtxR, overall 45 genomic targets were found across the tested conditions (Figure S5, Table S3). Representative extracted peaks for all genomic targets of DtxR are shown for the iron excess condition in Figure S6 and for the heme condition in Figure S7. Almost all are located in the upstream region of open reading frames. Among those, we could identify 20 out of the 54 targets, which were already described by previous studies using transcriptome analyses and EMSAs (Brune et al. 2006; Wennerhold and Bott 2006). The most prominent binding peak at both iron excess and heme conditions was found upstream of NCgl0484, encoding a Fe^3+^ siderophore transport system, which was already shown to be repressed by DtxR in vitro (Brune et al. 2006). Interestingly, additional 25 yet unknown targets bound by DtxR were identified via ChAP‐Seq (Figure S5). Those include, for example, the prophage gene NCgl1781, *metH* coding for the homocysteine methyltransferase, and *cepA* coding for a putative toxin efflux permease. Among these novel targets, 11 were detected under iron excess condition, and 17 targets were bound during growth on heme (Figure 2A), while only 3 targets were found in both conditions (NCgl1781, NCgl1376 and NCgl0193; genes encoding hypothetical proteins) (Table S3). Interestingly, also novel targets were found that were predicted by motif search in silico but could not be verified in vitro in previous studies (Wennerhold and Bott 2006). These include genes encoding the putative membrane protein *wzy*, the putative oxidoreductase dehydrogenase *oxiB*, the transcriptional regulator NCgl0176, and the tyrosine recombinase *xerC*. Remarkably, we observed the trend that peaks that are closer to the transcriptional start site featured higher peak intensities (Figure 2B).

#### 3.1.2 Condition‐Specific Analysis of HrrA Binding Revealed Robust Homeostasis Under Iron Excess and Heme Conditions

For the heme‐sensitive regulator HrrA, overall 332 targets were identified (Figure S5, Table S4), with 269 binding in upstream regions and 63 inside open reading frames. 122 out of 231 targets identified by former studies could be confirmed (Keppel et al. 2020). In line with previous studies, the most prominent peak for HrrA binding corresponds either to the myo‐inositol‐1 or 4‐monophosphatase *suhB* or polyphosphate glucokinase *ppgK* (Keppel et al. 2020). Additionally, 210 new targets were identified (40% in the iron excess conditions and 26% found solely during growth on heme). Among the new targets are, for example, the central carbon metabolism‐related targets *aceA, pyc*, or *tkt*, as well as genes involved in signal transduction, including *cgtR2, hrcA, hspR, pdxR*, or *rbsR*. In total, 83 genes are bound by HrrA only in the iron excess condition. Most targets were not identified in previous studies focusing on heme as an iron source (Keppel et al. 2020). This includes the genes *rpoB* encoding the beta subunit of the DNA‐directed RNA polymerase, *ohr* encoding a putative organic hydroperoxide resistance and detoxification protein, the universal stress protein encoded by *uspA3*, and the gene for the translation initiation factor IF‐3 *infC*. Also for HrrA, we observed higher peak intensities for targets located in proximity to the TSS (Figure 2B). These results for both regulators underscore the value of conditional genome‐wide analysis in providing comprehensive insights into the regulons of global transcriptional regulators.

### 3.2 Evaluation and Refining of Binding Motifs

ChAP‐Seq analysis provides genome‐wide insights in binding site variations, offering potential to refine motif identification. Therefore, analysis on the binding motif was accomplished using the MEME‐ChIP tool (Bailey and Elkan 1994) for both transcriptional regulators under iron and heme conditions.

Based on the previously predicted DtxR motif (TAGGTTAG(G/C)CTAACCTAA), the binding peak ChAP‐Seq results for the iron excess condition lead to the motif AGGDNAGSCTAACCTWAKTT (*E*‐value = 6.2 × 10^−12^), while the heme condition led to a similar motif GKVAGSCTHRCCTAASHWA (*E*‐value = 1.0 × 10^−12^) (Figure 2C). The predicted motifs show a significant overlap with the previously predicted motif and fit well with the DtxR motif of *C. diphtheriae* (Kunkle and Schmitt 2003). However, it became evident from our analysis that the palindromic consensus is less conserved than previously anticipated. Therefore, the predicted motif by Brune et al. (2006) was examined within the DtxR target sequences via the FIMO analysis tool (Grant et al. 2011). The alignments (Figure 2D) revealed the presence of the palindromic sequence across targets, but with considerable variation, especially for the so‐far unknown targets (Figure S8).

For HrrA, the found motif from iron excess CMAMCDAAAGKTKGA (*E*‐value = 2.4 × 10^−40^) and from heme condition CAWHCRAAAGDTKKA (*E*‐value = 9.9 × 10^−59^) also corresponds to the motif found by (Keppel et al. 2020), with an even more significant *E*‐value (Figure S9). These findings underscore the power of ChAP‐Seq for characterizing binding site variability and refining motif predictions for global transcriptional regulators under different environmental conditions.

### 3.3 Validation of Novel DtxR Targets

**FIGURE 3:**
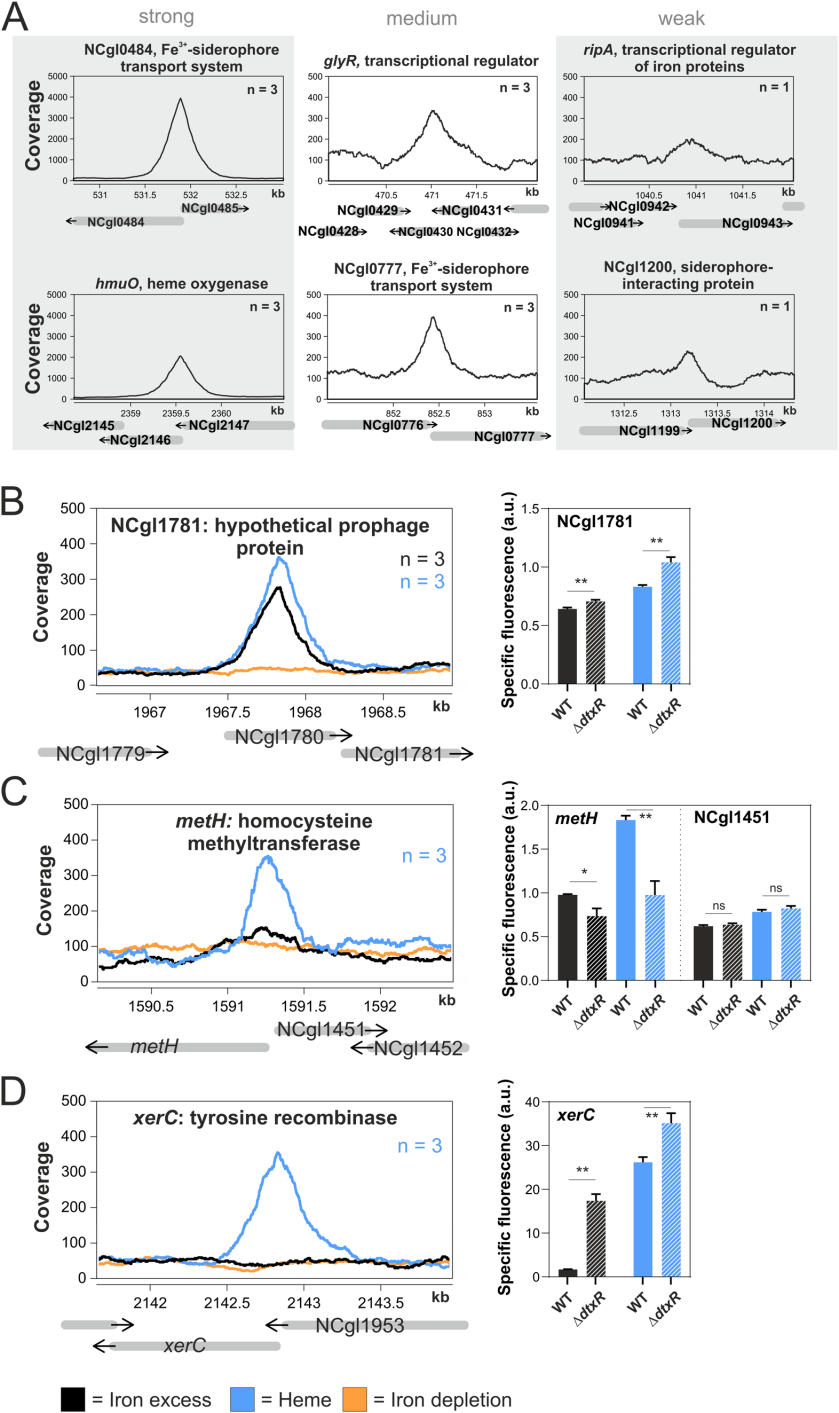
Binding peaks and reporter outputs of selected novel binding targets of DtxR. (A) Exemplarly, six known DtxR targets that were confirmed within this in vivo ChAP‐Seq experiment are shown featuring strong, medium and low binding peak intensities, respectively. (B‐D) Binding of DtxR to selected novel targets (NCgl01781, *metH* and *xerC*) under iron excess (black), heme (blue) and iron depletion (orange) conditions. *n* represents the number of replicates where a significant peak was identified. Additionally, bar plots represent the specific fluorescent reporter output after 2 h of either *Corynebacterium glutamicum* WT (filled bar) or a *dtxR* deletion strain Δ*dtxR* (striped bar) transformed with a reporter plasmid coupling the activity of the respective promoter region to *venus* expression. Statistical significance was confirmed by Student's t‐test (*p* ≤ 0.05).

Several already known DtxR targets have been confirmed in vivo by this ChAP‐Seq experiment, yielding different peak variants from strong, over medium to weak binding intensity (Figure 3A). Moreover, many so far unknown potential targets were identified. To confirm binding peaks and achieve a comprehensive understanding of gene regulation, it is essential to link DNA binding with gene expression data. This allows for the assessment of whether transcription factor binding to target sites is reflected in changes at the gene expression level. On this basis, a subset of novel targets of DtxR were chosen for further analysis, varying from peaks found in both iron excess and heme conditions, or only in one of them. For each target gene, a reporter plasmid was constructed in which the coding sequence for the fluorescent protein Venus was placed under the control of the respective DtxR‐targeted promoter region. All constructs were tested in *C. glutamicum* wild type and a Δ*dtxR* deletion strain at iron excess and heme conditions (Figure 3B–D, Figure S10).

The novel target NCgl1781 coding for a hypothetical prophage protein exhibited the highest peak intensity among novel targets and was confirmed to be repressed by DtxR under both conditions (Figure 3B). However, only minor fold changes were observed via reporter assays. In contrast, *metH* encoding homocysteine methyltransferase was activated by DtxR in both conditions, but promoter binding was consistently observed only in the heme condition (Figure 3C). The novel target *xerC*, encoding a tyrosine recombinase, was also exclusively identified under heme conditions via ChAP‐Seq but was clearly repressed by DtxR in both conditions (Figure 3D). Additional targets showed mixed responses, like *gyrB* coding for the DNA topoisomerase/gyrase IV (Figure S10A) which displayed a binding peak only in the iron condition but changes in reporter assays only in the heme condition. However, *gyrB* is likely a target underlying complex regulation involving further transcriptional regulators as well as DNA topology. In contrast, *wzy* coding for a putative membrane protein was not significantly influenced by DtxR (Figure S10B). Further targets were confirmed by this approach, including *cepA* (putative toxin efflux permease), as well as NCgl0177 (putative membrane protein) and NCgl2776 (putative secreted protein) (Figure S10C–E). Furthermore, gel shift assays confirmed DtxR binding to the promoter regions of *cepA*, NCgl2776, *xerC*, and *metH*, while no binding to NCgl0177 and *gyrB* could be detected in vitro (Figure S11). Overall, we identified several previously unknown DtxR targets that had not been detected under standard conditions or in in vitro binding assays.

### 3.4 DtxR And HrrA Regulons Are Tightly Interconnected

**FIGURE 4:**
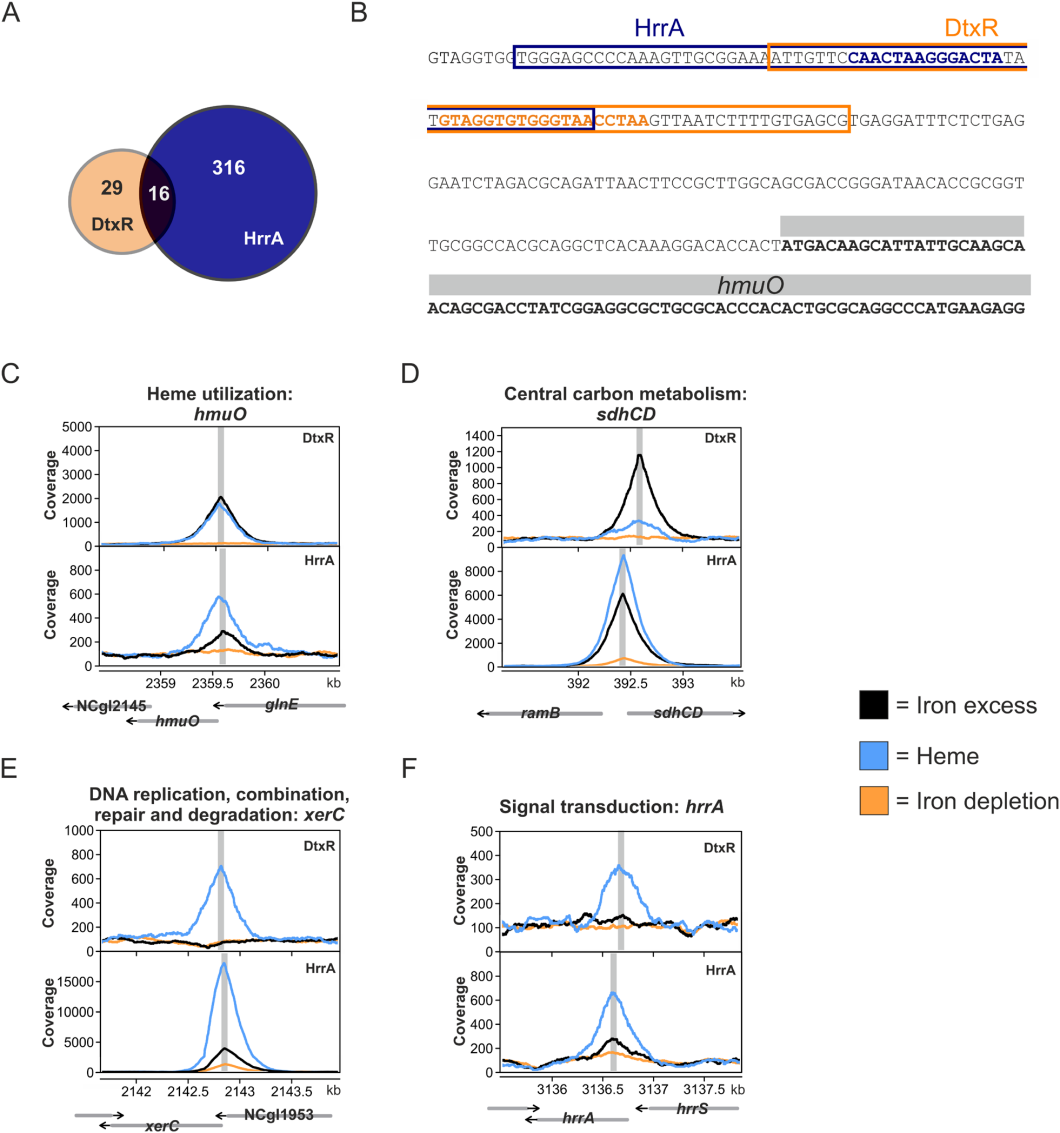
Shared targets of the regulators DtxR and HrrA show interconnection between iron and heme regulatory networks. (A) Quantitative overview of shared targets regulated by DtxR and HrrA. (B) Upstream region of *hmuO* (grey). Predicted binding regions for DtxR and HrrA are indicated by orange and blue boxes. The sequence corresponding to the actual binding motif as revealed by FIMO analysis (Grant et al. 2011) is highlighted in the respective color. (C–F) Peak detection in the region of *hmuO, sdhCD, xerC*, and *hrrA*, respectively, with gene locations represented by grey arrows. Binding peaks are shown for iron excess (black), heme (blue) and iron depletion (orange). Grey vertical lines mark the peak maxima.

For the first time, our genome‐wide profiling of HrrA and DtxR enables a direct comparison of their binding patterns on shared targets, offering new insights into potential regulatory interference. Overall, 16 targets, which were regulated by both DtxR and HrrA, could be identified (Figure 4A, Table S5). Examples of binding peaks are given in Figure 4C–F, with their genomic context presented additionally in Figure 4B and Figure S12. A notable example of regulatory interference between DtxR and HrrA is observed in the promoter region of the heme oxygenase *hmuO*. The locations of DtxR and HrrA binding for *hmuO* regulation are in close proximity to each other, with significant overlap and their motifs separated only by 3 bp (Figure 4B,C). While DtxR acts as a repressor of *hmuO*, HrrA was shown to be crucial for activating *hmuO* expression (Frunzke et al. 2011; Keppel et al. 2019). For this particular example, we observed a higher coverage of HrrA on heme and a slightly higher coverage of DtxR on iron. A similar trend—with higher coverage for DtxR on iron and HrrA on heme—was also observed for *sdhCD* (Figure 4D). Here, however, the binding sites are separated by ~100 bp, speaking against a direct interference effect. Overall, we observed a trend of higher DtxR peak intensities under iron conditions, while HrrA exhibited greater binding coverage under heme conditions (Figure S13). However, this overall trend did not establish when considering all shared targets. For example, DtxR binding to *xerC* was only observed in the presence of heme, while HrrA also showed a higher coverage under heme conditions (Figure 4E). However, this does not necessarily mean that these targets are not affected by DtxR regulation under the other condition (Figure 3D), but binding was only observable in ChAP‐Seq experiments in that case.

Further, we could confirm binding of HrrA to itself, activating the expression of its own gene (Keppel et al. 2020) (Figure 4F). While caution is necessary when directly comparing peak intensities between conditions due to differences in regulator proteins and purification methods, it was observed that HrrA binding to its own promoter increased at rising heme levels. The DtxR binding site upstream of *hrrA* is slightly upstream, but with no overlap with the HrrA motif as for *hmuO*. Surprisingly, no significant DtxR binding could be detected under iron excess, showing the high sensitivity to slight fluctuations in cultivation conditions or experimental setups.

Overall, no consistent pattern of interference between DtxR and HrrA at the promoter regions of shared targets is observed, underscoring the complexity of regulatory networks that extend beyond the interaction of just two global regulators.

### 3.5 Weak Binding Sites Should Not Be Ignored

**FIGURE 5:**
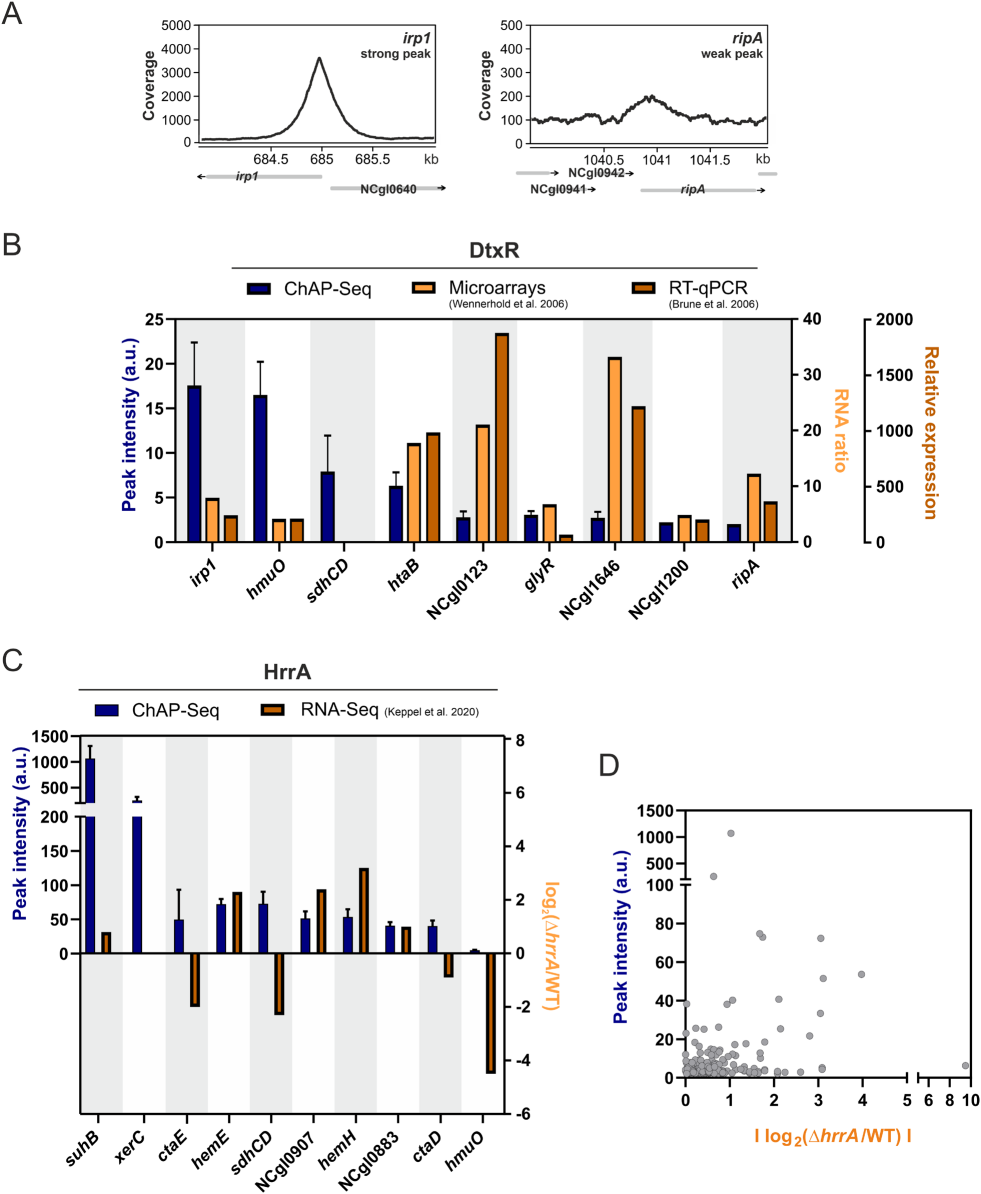
Weak binding targets should not be ignored—Correlation of ChAP‐Seq peak intensity and differential gene expression. (A) Exemplarly depiction of a strong DtxR binding peak (*irp1*, iron excess condition) versus a weak binding peak (*ripA*, iron excess condition). (B) Peak intensities of the ChAP‐Seq for DtxR under iron excess conditions (dark blue) were compared to the RNA ratio under iron excess (Δ*dtxR*/wild type) from a microarray analysis (Wennerhold and Bott 2006) (dark orange) as well as to the relative expression under iron excess in the deletion strain Δ*dtxR* compared to wild type analyzed via RT‐qPCR (Brune et al. 2006) (light orange). (C) Peak intensities of the ChAP‐Seq of HrrA in the presence of heme (dark blue) was compared to the log_2_(Δ*hrrA*/wild type) obtained from the time‐resolved RNA‐seq in the presence of heme (Keppel et al. 2020) (shades of orange) for a selection of targets with highest and lowest peak intensities. (D) 2D‐scatterplot showing no significant correlation between ChAP‐Seq peak intensities of HrrA in the presence of heme and the absolute value of log_2_(Δ*hrrA*/wild type) obtained from the time‐resolved RNA‐seq data under heme conditions (Keppel et al. 2020) for all identified targets.

ChAP‐Seq experiments often produce a large number of peaks with a weak binding coverage (Figure 5A). To address their physiological relevance, we correlated peak intensities of ChAP‐Seq experiments with microarray, RT‐qPCR, and RNA‐Seq data obtained previously (Brune et al. 2006; Keppel et al. 2020; Wennerhold and Bott 2006). Figure 5B compares DtxR peak intensities with differential gene expression levels (Δ*dtxR*/WT) obtained from microarray analysis (Wennerhold and Bott 2006) and RT‐qPCR results (Brune et al. 2006) for the targets identified in all three studies under iron excess conditions.

Interestingly, this analysis revealed an anti‐proportional trend of peak intensities and differential gene expression determined by different methods. The highest binding peaks were found in this study for *irp1* and *hmuO*, where differential gene expression levels were shown to be rather low when comparing expression in *C. glutamicum* wild type with a mutant lacking *dtxR*. Conversely, targets like NCgl0123 (hypothetical protein) and NCgl1646 (putative secreted hydrolase in the prophage CGP3 region) displayed high differential expression despite low binding coverage in ChAP‐Seq experiments.

A similar trend was observed for HrrA (Keppel et al. 2020), where several targets with only weak binding coverage featured strong changes at the level of gene expression when comparing *C. glutamicum* wild type with a mutant lacking *hrrA* (Figure 5C). For example, *suhB* displayed the highest binding peak but one of the lowest log_2_(Δ*hrrA*/WT) ratios, whereas *hmuO* showed the opposite: a low peak intensity but high differential expression in the absence of HrrA. While the correlation between peak intensities and gene expression is not consistently strong across all peaks (Figure 5D), this analysis underscores that weak binding peaks should not be dismissed as false positives too early, as they may hold significant biological relevance.

## 4 Discussion

In this study, we performed a genome‐wide profiling of the global transcriptional regulators DtxR and HrrA coordinating iron and heme homeostasis in *C. glutamicum* . The obtained results provide valuable insights into the interaction and interference of iron and heme regulatory networks and demonstrate the robust homeostasis conferred by the underlying strategies. Genome‐wide binding analysis revealed significant differences in binding patterns under varying iron conditions, using FeSO_4_ or heme as iron sources. This highlights the potential of genome‐wide studies performed under different iron regimes in uncovering a substantial number of previously unknown potential targets for both regulators. The number of targets identified for DtxR and HrrA in this study aligns closely with the predicted contributions of these regulators to the *C. glutamicum* regulatory network, accounting for approximately 3% and 21%, respectively (relation percentage: 3/21 = 0.143; relation targets: 45/332 = 0.136) (Escorcia‐Rodríguez et al. 2020). It is important to note that the altered binding pattern of DtxR under heme conditions is primarily due to iron released by the heme oxygenase HmuO (Figure 1A), rather than direct binding of heme to DtxR. It is also likely that *C. glutamicum* possesses additional, yet unidentified, mechanisms for liberating iron from heme beyond HmuO (Kunkle and Schmitt 2007), resulting in a markedly different iron availability compared to conditions with pure iron excess. Furthermore, the heme‐responsive regulator HrrA—whose regulon partially overlaps with that of DtxR—exerts additional regulatory influence on DtxR targets in the presence of heme.

In this in vivo study, we successfully confirmed many previously known DtxR targets from the literature. The absence of certain targets identified in earlier in vitro studies (Brune et al. 2006; Wennerhold and Bott 2006) may be attributed to false‐negatives; however, alternative explanations are plausible. The discrepancies could arise from conditional effects, such as differences in regulatory dynamics over time, variations in cultivation conditions, or transcription factor concentrations. Recent ChIP‐Seq analysis of Fur, which is a functional homolog to DtxR in many Gram‐negative bacteria, demonstrated a graded response of this transcriptional regulator in *Bacillus subtilis* , caused by different protein‐DNA‐binding affinities (Pi and Helmann 2017). With decreasing iron concentrations Fur derepressed its targets stepwise in ‘three waves’. Follow‐up studies even determined additional targets for this well‐characterized global regulator (Pi and Helmann 2018). This highlights the importance of binding analyses at different cultivation conditions to fully characterize the highly dynamic binding behavior of global transcriptional regulators. Another reason could be a more broadly interaction and interference with nucleoid‐associated proteins or other transcription factors besides HrrA that mask DtxR binding (Myers et al. 2013; Pfeifer et al. 2016), which potentially results in observing binding to different extents at the different conditions. However, a more simple explanation could be that the other sites are too weak in binding due to, for example, hardly accessible binding sites and therefore cannot be detected with this experimental setup. Moreover, it is important to note that the HrrA analysis in this study used only one harvesting time point (2 h vs. 0, 0.5, 2, 4, 9 and 24 h), but investigated the effect of different iron sources (Keppel et al. 2020). As stated in the previous study, HrrA regulation is highly dynamic, characterized by pronounced temporal changes. These variations underline the sensitivity of such experimental setups, particularly regarding factors such as timing and sample handling (Chen et al. 2012; Myers et al. 2015; Myers et al. 2013) and condition‐dependent regulatory dynamics.

Several genes, including the newly identified targets *metH* and *xerC*, were identified to be bound by DtxR exclusively under heme conditions. The gene *metH* encodes the homocysteine methyltransferase, which catalyzes the last step of methionine synthesis from homocysteine. Within this study, DtxR was demonstrated for the first time as an activator of *metH* expression in Corynebacteria. *metH* was already known to be regulated by the methionine and cysteine biosynthesis repressor McbR (Rückert et al. 2003). For DtxR of *C. diphtheriae* , binding to methionine related genes *metA* and *mapA* was also predicted (Yellaboina et al. 2004). Interestingly, Fur, the master regulator of iron in *E. coli* , was likewise shown to regulate methionine biosynthesis via the gene *metH* (Stojiljkovic et al. 1994). Methionine can function as an antioxidant due to its high susceptibility to oxidation, forming methionine sulfoxide (Levine et al. 1996; Slyshenkov et al. 2002). This oxidized form can be reduced back to methionine by methionine sulfoxide reductases (Brot and Weissbach 1983), a process that also occurs in *C. glutamicum* via the action of the reductase MsrA (Si et al. 2015). Subsequently, a regulatory connection between oxidative stress induced by elevated iron levels and methionine synthesis could be a valuable strategy for ROS counteraction and should be addressed in future studies. In a broader context, early studies already demonstrated that methionine promotes the catalytic activity of the heme oxygenase and ferritin in endothelial cells, thereby suppressing free radical formation (Erdmann et al. 2005). This connection highlights the potential for methionine to play a broader role in oxidative stress mitigation and iron homeostasis.

The repressed target *xerC*, encoding a tyrosine recombinase, was previously suggested by motif prediction, but could not yet be verified in vitro (Wennerhold and Bott 2006). Tyrosine recombinases are responsible for site‐specific DNA recombination resulting in a variety of genetic rearrangements, e.g., integration, excision, or inversion via breaking and rejoining single strands (Grindley et al. 2006). XerC and XerD recombinases are highly conserved in many bacteria. In *E. coli*, they were shown to aid in proper plasmid and chromosome segregation during cell division, dependent on the DNA‐translocase FtsK (Aussel et al. 2002; Blakely et al. 1991). Contrary to this, the functional DtxR‐homolog Fur was not described to regulate *xerC* in *E. coli* (Seo et al. 2014). The repression of *xerC* expression by DtxR suggests a potential mechanism for halting DNA recombination events under high iron conditions, possibly to prioritize DNA repair in response to iron‐induced oxidative stress. Notably, *xerC* has also been shown to be repressed by HrrA, highlighting a possible convergence of regulatory control by these two global regulators (Keppel et al. 2020). DtxR also controls several genes located in prophage elements (Frunzke et al. 2008) and XerC‐like proteins were reported to play a role in phage‐mediated recombination events (e.g., prophage integration). Therefore, this regulation might also link the activity of mobile genetic elements to oxidative stress levels.

Another novel target within the prophage region CGP4 is NCgl1781, which is repressed by DtxR (Frunzke et al. 2008; Helfrich et al. 2015; Pfeifer et al. 2016). Previous studies identified further DtxR targets within the large prophage CGP3 (Wennerhold and Bott 2006), while it was also shown that the induction of this prophage is triggered by iron‐mediated oxidative stress (Boeselager et al. 2018; Frunzke et al. 2008). These results indicate the intricate integration of these viral elements into host regulatory networks enabling them to “eavesdropping” on cellular stress responses to modulate their activity. However, many of those prophage genes regulated by DtxR encode hypothetical proteins, requiring further investigation to elucidate their specific functions and regulatory roles.

The majority of new HrrA targets have been identified under conditions of iron excess, i.e., a condition not explored in previous studies. Many of these targets are hypothetical proteins, as well as genes associated with translation and central carbon metabolism. Further validation of these targets by enlarging transcriptomic data for the iron condition as well as the particular time point of 2 h is essential to assess the impact of this large set of targets.

Overall, we did not observe a consistent pattern of interference between DtxR and HrrA. However, the interaction between these two global iron‐related regulators represents just one facet of a much more intricate regulatory network involving numerous additional factors. Recent studies in *Caulobacter crescentus* have examined the genome‐wide binding pattern of IscR in comparison to Fur, the respective key regulators of iron homeostasis. These investigations identified several interference effects between these two regulators, while further suggesting the involvement of additional players (Dos Santos et al. 2024). This is additionally highlighted by previous ChIP‐Seq studies on *Mycobacterium tuberculosis* , where the combination of binding profiles of in total 50 transcription factors has provided transformative insights into regulatory interferences, networks, and finally pathogenesis (Galagan et al. 2013). Combining such manifold genome‐wide binding data with further expression analyses can significantly broaden our knowledge on regulation, as demonstrated in numerous studies across domains of life (MacPhillamy et al. 2022; Qin et al. 2022; Rustad et al. 2014).

In general, it should be noted that binding affinities and strengths are not necessarily reflected by peak intensities (Myers et al. 2013), but are further influenced by a wide range of factors in vivo, including inter‐ and intramolecular interactions (e.g., electrostatic or hydrophobic forces), DNA topology, and interference with other DNA‐binding proteins (Dorman 2019; Ponta et al. 1992; Sims et al. 2005). This study further highlighted that we cannot drive simple conclusions from peak intensities to the impact of regulator binding on the level of gene expression. Weak binding peaks should not be dismissed, as they may correspond to targets that are strongly influenced by the respective transcriptional regulator.

## 5 Conclusion and Future Perspectives

This study provides comprehensive insights into the genome‐wide binding profiles of the two global transcriptional regulators, DtxR and HrrA. Our findings highlight the potential for the discovery of novel regulatory targets through profiling under different environmental conditions, specifically using iron or heme as iron sources. The results underscore the well‐orchestrated homeostasis maintained by DtxR and HrrA, with similar binding patterns observed across conditions, likely reflecting comparable but not identical intracellular pools of Fe^2+^ and heme. Importantly, our data reveal that iron‐related regulatory networks are more intricate than a simple interplay between these two global regulators. The observation that targets with low ChAP‐Seq peak intensities often exhibit strong regulatory outcomes suggests that even low‐coverage targets should not be disregarded, as they may have significant biological relevance. In summary, the reported data expand our understanding of the global regulatory network governing iron homeostasis and emphasize that the relation between binding behavior and functional or structural impact of prokaryotic transcription factors exists along a continuum.

## Author Contributions

**Aileen Krüger:** conceptualization, investigation, writing – original draft, methodology, validation, writing – review and editing, visualization, supervision, formal analysis. **Ulrike Weber:** methodology, investigation. **Julia Frunzke:** conceptualization, investigation, writing – review and editing, funding acquisition, writing – original draft, supervision.

## Ethics Statement

The authors have nothing to report.

## Consent

The authors have nothing to report.

## Conflicts of Interest

The authors declare no conflicts of interest.

## Supporting information


Data S1.

Table S3.

Table S4.

## Data Availability

The datasets generated and/or analyzed during the current study are included in this published article and its supplementary information files. Further data, including sequencing reads as well as ChAP‐Seq analyses, can be found in the ARC Data Hub https://archive.nfdi4plants.org/records/14s2v‐ztq25. It is available under https://doi.org/10.60534/14s2v‐ztq25. Further, sequence data have been submitted to the European Nucleotide Archive (ENA) at EMBL‐EBI under accession number PRJEB83556 (https://www.ebi.ac.uk/ena/browser/view/PRJEB83556). The custom‐developed software adapted in this study for ChAP‐Seq analysis is publicly available at the GitHub repository under the link https://github.com/afilipch/afp.
