# Supplementary material for: Genome‐Wide Analysis of DtxR and HrrA Regulons Reveals Novel Targets and a High Level of Interconnectivity Between Iron and Heme Regulatory Networks in Corynebacterium glutamicum
Source: Mol Microbiol. 2025 May 16;124(2):115–30. doi: 10.1111/mmi.15376 (PMC12327846; doi:10.1111/mmi.15376)
Supplement: Supplementary file 1 — Data S1. [file MMI-124-115-s002.pdf]

Supplements to: Genome-wide analysis of DtxR and HrrA regulons reveals novel targets and a high level of interconnectivity between iron and heme regulatory networks in *Corynebacterium glutamicum*

Aileen Krüger[1], Ulrike Weber[1], Julia Frunzke[1*]

[1] Forschungszentrum Jülich GmbH, Institute for Bio- and Geosciences 1, IBG1, 52425 Jülich, Germany

***Correspondence:**
Corresponding Author
j.frunzke@fz-juelich.de

Figure S1: Analysis of a *C. glutamicum* strain with a tagged DtxR variant.

Figure S2: ChAP-Seq procedure for genome-wide profiling of DtxR and HrrA DNA-binding in *C. glutamicum.*

Figure S3: Further replicates of genome-wide profiling of DtxR and HrrA DNA-binding in *C. glutamicum*.

Figure S4: Cryptic peaks in iron depletion sequencing run.

Figure S5: Numeric summary on targets of DtxR and HrrA.

Figure S6: Genomic targets bound by DtxR during growth under iron excess conditions.

Figure S7: Genomic targets bound by DtxR during growth under heme conditions.

Figure S8: Motif alignment for DtxR binding as predicted from ChAP-Seq data ($p > 1.0e^{-05}$).

Figure S9: HrrA Motif predicted from ChAP-Seq results.

Figure S10: Binding peaks and reporter outputs of further selected novel DtxR targets.

Figure S11: In vitro DtxR binding to selected weak targets identified by ChAP-Seq.

Figure S12: Location of DtxR and HrrA peaks in the promoter region of selected shared target genes.

Figure S13: Ratios of iron and heme peak intensities.

Table S1: Bacterial strains and plasmids used in this study.

Table S2: Oligonucleotides used in this study provided by Eurofins Genomics (Germany).

Table S3: Complete dataset of genome wide DtxR binding (ChAP-Seq) at iron excess (100 µM $FeSO_4$) and heme (4 µM heme) conditions in triplicates. (external Excel file)

Table S4: Complete dataset of genome wide HrrA binding (ChAP-Seq) at iron excess (100 µM $FeSO_4$) and heme (4 µM heme) conditions in triplicates. (external Excel file)

Table S5: Overlapping peaks for DtxR and HrrA.

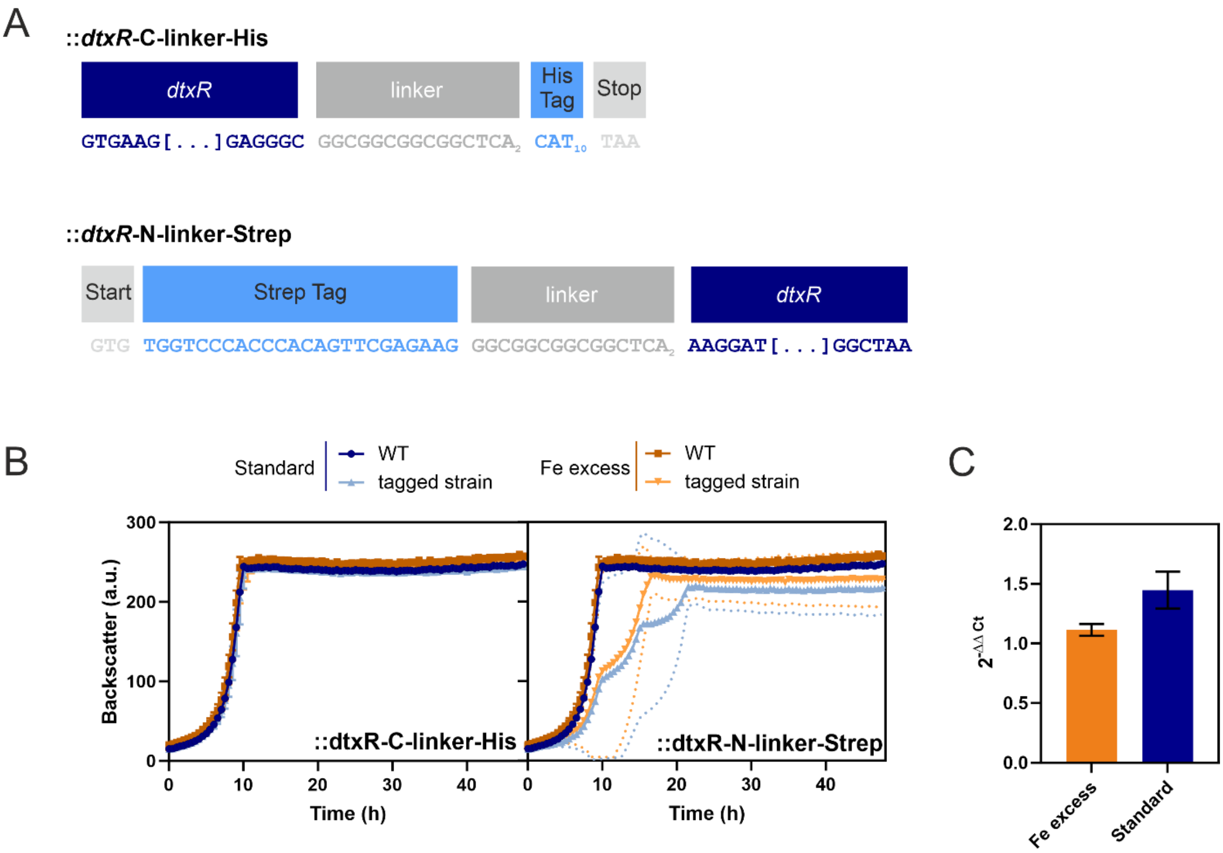

Figure S1: Analysis of a *C. glutamicum* strain with a tagged DtxR variant. (A) Schematic representation of the tagged DtxR variants ::*dtxR*-C-linker-His (top) and ::*dtxR*-N-linker-Strep (bottom). (B) *C. glutamicum* wild type as well as the strain WT::*dtxR*-C-linker-His with a C-terminally His-tagged DtxR variant (left) and the strain WT::*dtxR*-N-linker-Strep with an N-terminally Strep-tagged DtxR variant (right) were inoculated to a starting-OD_{600} of 1 in CGXII medium containing 2% glucose and either the standard amount of iron (36 µM $FeSO_4$) or iron excess (100 µM $FeSO_4$) in the BioLector® microtiter cultivation system of Beckman Coulter GmbH (Baesweiler) (Kensy et al., 2009). Data represents the average of three biological replicates including standard deviations depicted as error bars. (C) The WT::*dtxR*-C-linker-His strain was further analyzed in a qPCR experiment. Data represents two biological and three technical replicates. Fold-change was calculated according to the $2^{-\Delta\Delta Ct}$ (Livak and Schmittgen, 2001).

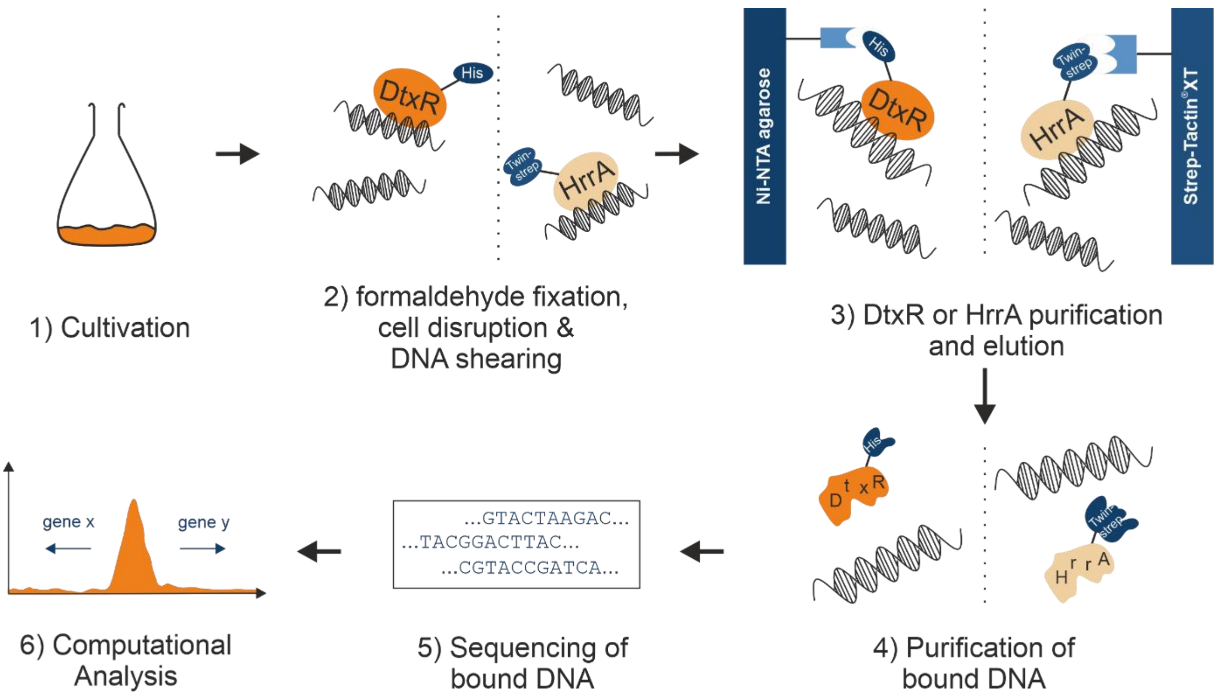

Figure S2: ChAP-Seq procedure for genome-wide profiling of DtxR and HrrA DNA-binding in *C. glutamicum.* (A) Schematic representation of the chromatin affinity purification sequencing (ChAP-seq) experimental setup for both a DtxR His-tagged and a HrrA twin-Strep-tagged variant. Cultivation was performed in minimal medium CGXII supplemented with 2% glucose and either 100 µM $FeSO_4$, 4 µM heme or no iron source.

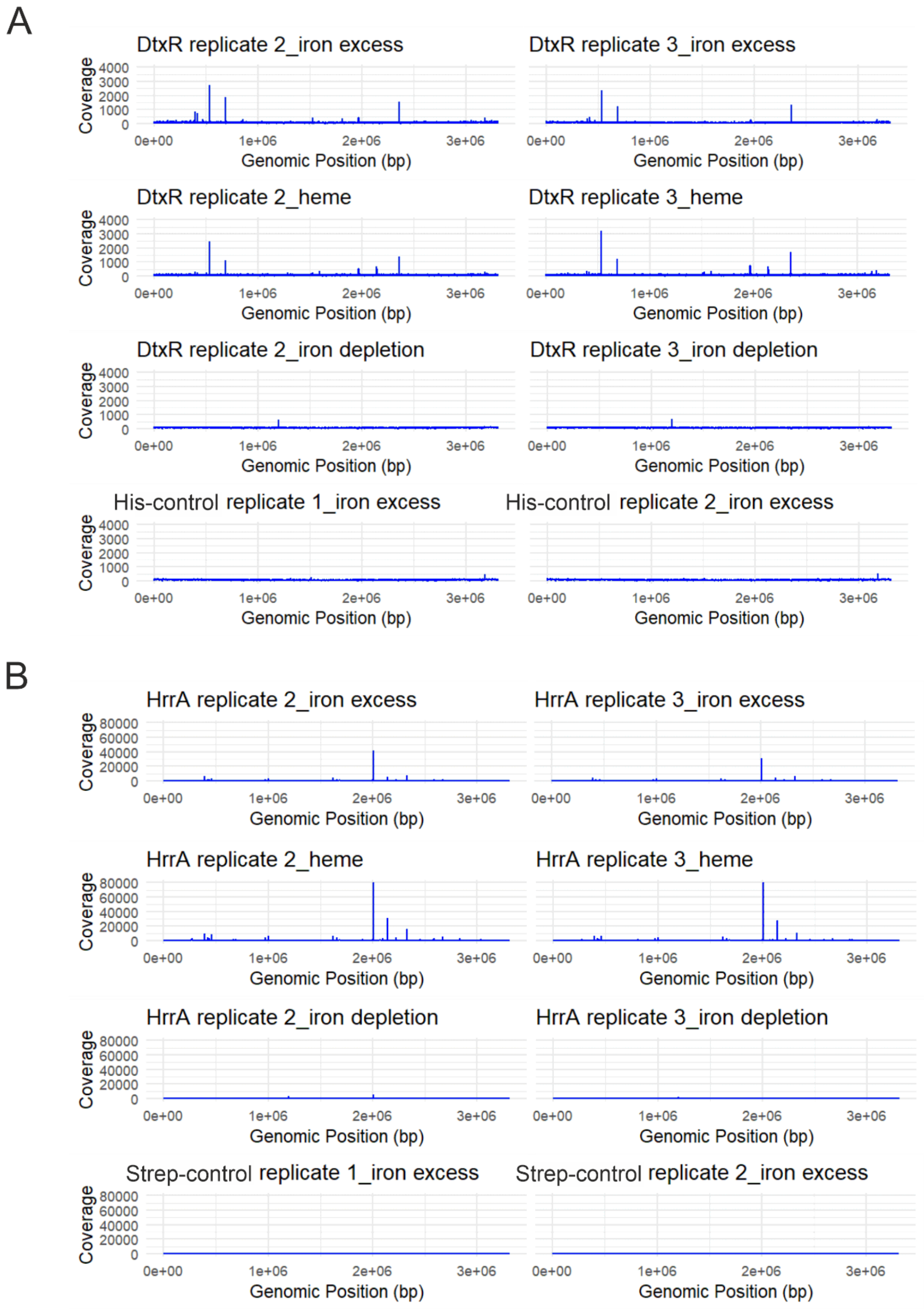

Figure S3: Further replicates of genome-wide profiling of DtxR and HrrA DNA-binding in *C. glutamicum*. Mapping of ChAP-seq reads for (A) DtxR and (B) HrrA to the *C. glutamicum* ATCC 13032 genome NC_003450.3. DNA was obtained by affinity purification of DtxR and HrrA from cultures grown under iron excess (100 µM $FeSO_4$), heme (4 µM heme) or iron depletion (0 µM $FeSO_4$). Control: two replicates were performed with the wild type (WT) possessing non-tagged regulator variants as negative control according to His- or Strep-tag protocol, respectively. No significant background signal was obtained for the control samples.

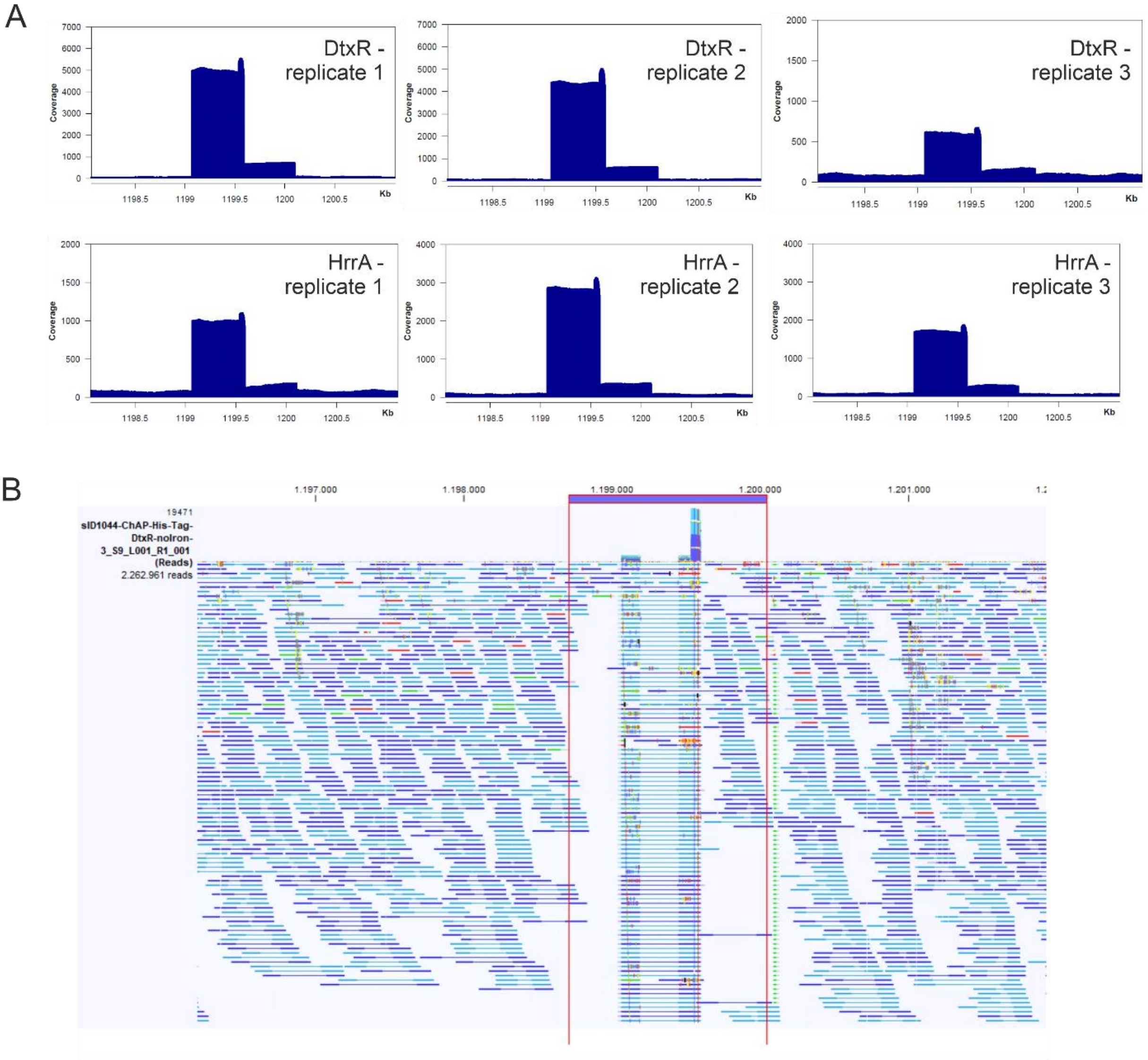

Figure S4: Cryptic peaks in iron depletion sequencing run. (A) Cryptic peak found in every sample of this sequencing run at the same position, presumably due to technical sequencing issue. (B) Read zooming-in (CLC Workbench 20.0.4) demonstrating the high amount of same cryptic reads. Based on these results, it can be summarized that this does not represent a binding peak of the underlying transcriptional regulators.

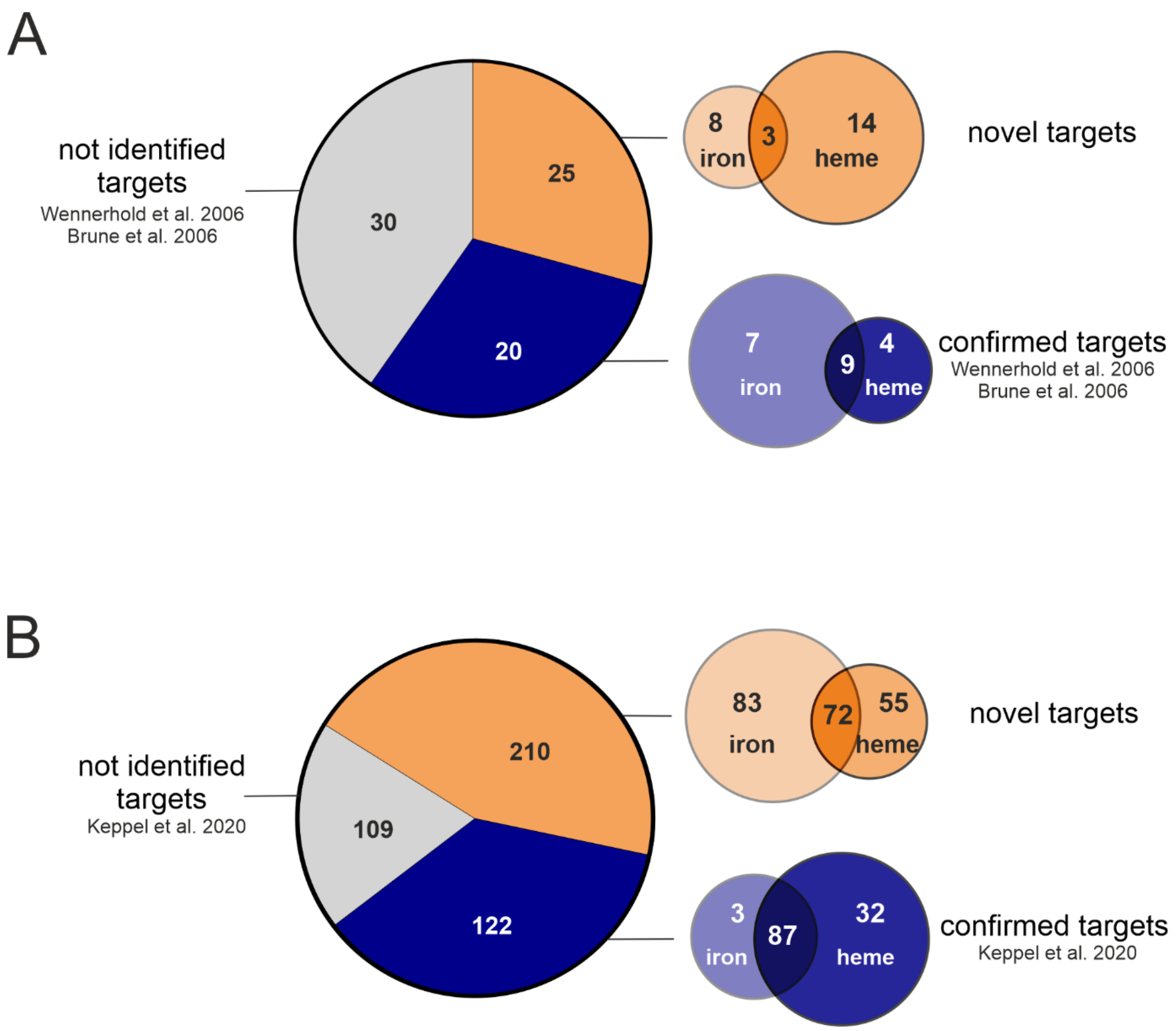

Figure S5: Numeric summary on targets of DtxR and HrrA. (A) Pie charts comparing targets of DtxR that are new, already confirmed by previous studies and predicted from previous studies (Brune et al., 2006; Wennerhold and Bott, 2006) but not found in this ChAP-Seq splitting up based on conditions. Total = 75. (B) Pie charts comparing targets of HrrA that are new, already confirmed by previous studies and predicted from previous studies (Keppel et al., 2020) but not found in this ChAP-Seq splitting up based on conditions. Total = 441.

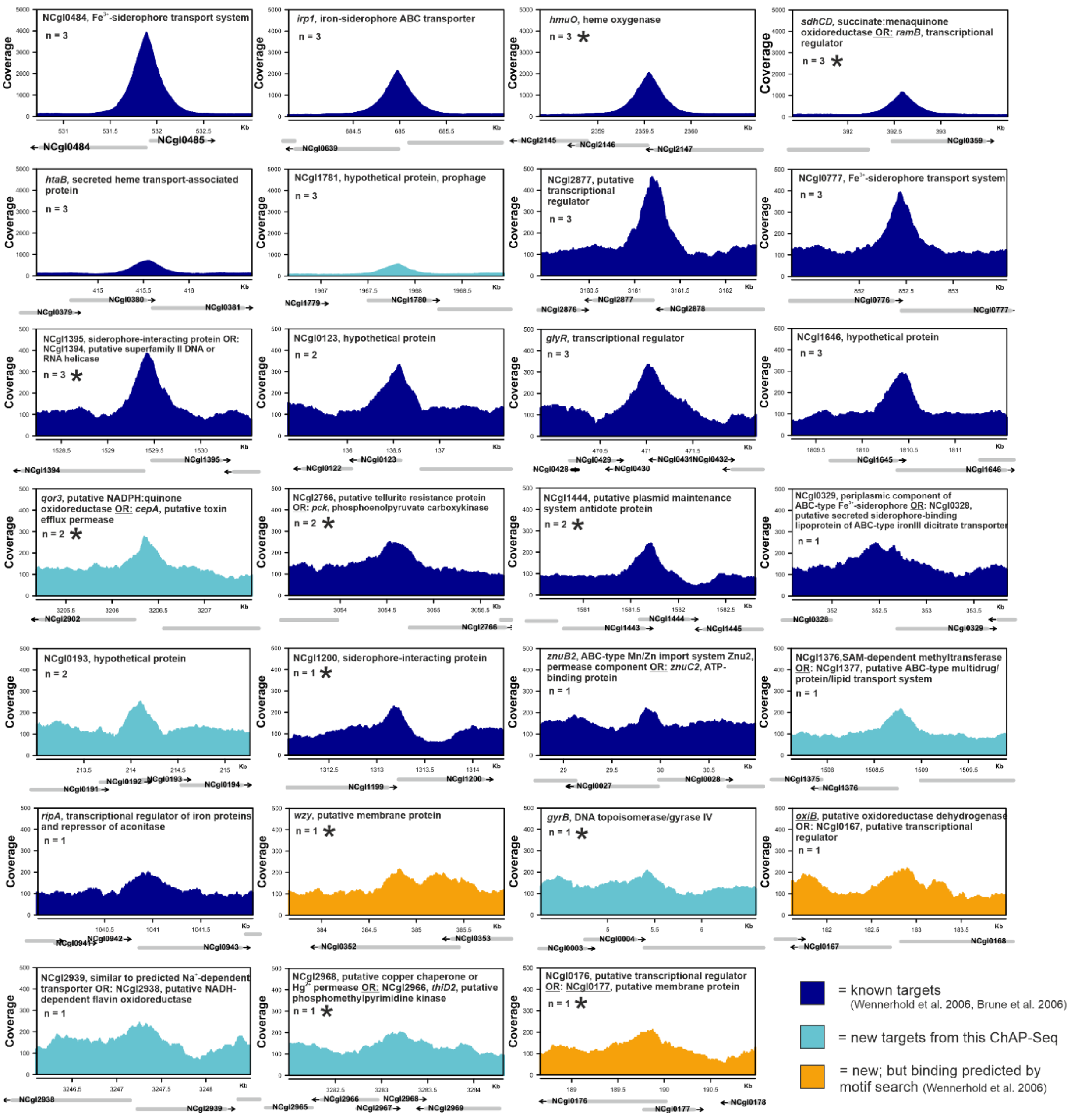

Figure S6: Genomic targets bound by DtxR during growth under iron excess conditions. Coverage of binding peaks (y-axis) plotted against the respective genomic regions of *C. glutamicum* ATCC 13032 (x-axis), genes are indicated below with grey arrows. Peaks include known targets (dark blue; Wennerhold and Bott (2006) and Brune et al. (2006)) novel targets identified in this study (light blue), and predicted targets of previous studies (orange). The number of replicates where a significant peak could be found is shown in each respective graph as 'n'. '*' marks those targets which are shared with HrrA. In some cases ('OR'), regulator binding could affect expression of both divergent genes.

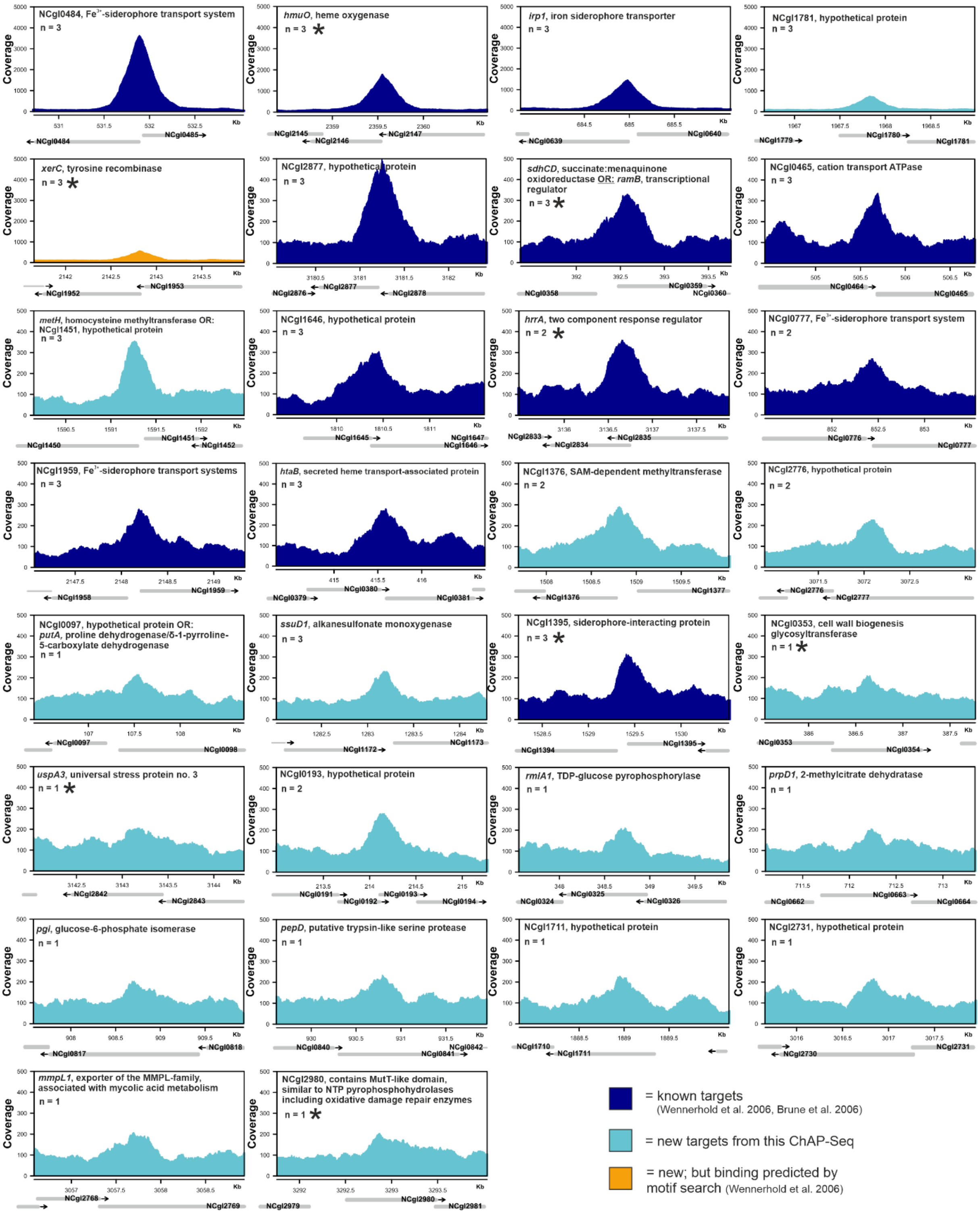

Figure S7: Genomic targets bound by DtxR during growth under heme conditions. Coverage of binding peaks (y-axis) plotted against the respective genomic regions of *C. glutamicum* ATCC 13032 (x-axis), genes are indicated below with grey arrows. Peaks include known targets, identified under iron excess conditions (dark blue; Wennerhold and Bott (2006) and Brune et al. (2006)) novel targets identified in this study (light blue), and predicted targets of previous studies (orange). The number of replicates where a significant peak could be found is shown in each respective graph as 'n'. '*' marks those targets which are shared with HrrA. In some cases ('OR'), regulator binding could affect expression of both divergent genes.

	Sequence (positions 5, 10, 15 marked)	p-value
predicted	T T A G G T T A G S C T A A C C T A A	
NCgl2766	T T A A C T T T G C C C T A C C T A A	2.01e-05
NCgl2877	G T C G G T A A G T C T T G C C A A A	0.000172
NCgl0465	T A G G G A A A G C C C A T C C T T A	0.000264
NCgl1444	T T A G G G A C G C T T T A C C T G C	0.000378
NCgl1781	T T G C T G C A G C C T A T C C A A A	0.000587
cepA	A T A A C G C A C C C T A A C C T T A	0.000801
qor3	T A A G G T T A G G G T G C G T T A T	0.000801
NCgl2731	T T G G G T T G C G G T A A T A A A G	0.00385
wzy	T C C G G T T G A A A A A C C C C A A	0.00493
NCgl0097	A A A G G A A A G C G T G C C C A A T	0.00631
NCgl2980	C C A G A A C A G C C A A A A C T C C	0.00834
oxiB	G C A T G G C A G G C A A A G C A C A	0.00834
NCgl0329	T G C C T C T G G C C A A A C C T G C	0.00834
NCgl0353	T G A A G G A A G C C G G A C A T T C	0.00834
NCgl0329	G C A G G T T T G G C C A G A G G C A	0.00834
NCgl0193	T A A G G T A C C C C T C A T G A A G	0.0113
rmlA1	G T A G G T G A G G T T G A T G C C C	0.0113
metH	T C A A C G C C G C C T A A G C A A C	0.0113
ripA	T G T A G T G A G G A T G A G A T A T	0.0136
znuB2	A T G A G T G A G C A T A A A A T T G	0.0136
ssuD1	A C C G C T T A G T C T T T T C C A G	0.0164
NCgl1711	A A A T G T G G A A C C A A C T A A A	0.0198
uspA3	C T T C G T T A A T C T T T T C C A G	0.0198
NCgl1376	T T A G A T A G G T A T A A A A A C C	0.0229
NCgl0664	C T T G G T G G G C A C G A T C G A C	0.0252
pepD	G C A G T T T T G C C T T C T G T C G	0.0324
NCgl2939	G T C A T T G T G G C G A T C A T C A	0.0324
NCgl2968	T G A G G G A G A G A G T A C T T C A	0.0324
gyrB	T T A A A A G T G G C A A A C A C T G	0.0324
NCgl0176	T T T C G G T A G C A C G G T T T A G	0.0396
NCgl1200	G G G G G T G C C T C T T A C G C A T	0.0464
hrrA	T C T G G T G G C T C C A C C G G C A	0.0464
pgi	T C G C G A C A G C C T C T T C A C C	0.0656
mmpL1	G A A G A T C A G C T C C A T C C G G	0.0798

Figure S8: Motif alignment for DtxR binding as predicted from ChAP-Seq data (p > 1.0e^{-05}). MUSCLE (Multiple Sequence Comparison by Log-Expectation) alignment (Madeira et al., 2024) of motifs found based on the predicted DtxR motif (Brune et al., 2006) throughout ChAP-Seq targets. Depicted are only targets where the motif hit corresponds to a p-value > 1.0e^{-05}, while those with lower p-values can be found in the main text, Figure 2D. Shades of blue represent the percentage identity. Gene names depicted in light blue are those that are potential novel targets, unknown so far.

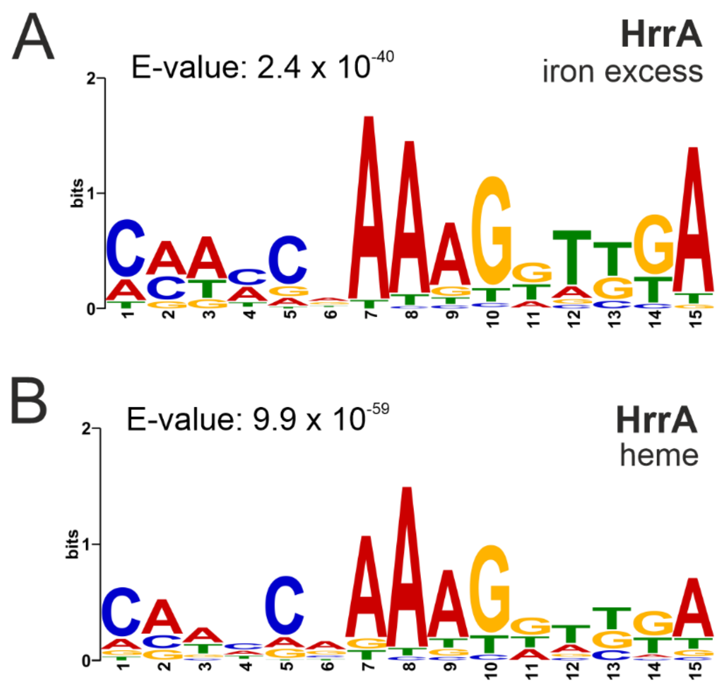

Figure S9: HrrA Motif predicted from ChAP-Seq results. MEME-ChIP prediction of the HrrA binding motif based on ChAP-Seq binding peaks extracted for (A) the iron excess condition and (B) the heme condition (Bailey and Elkan, 1994). The motifs fit to the previously reported HrrA motif (Keppel et al., 2020).

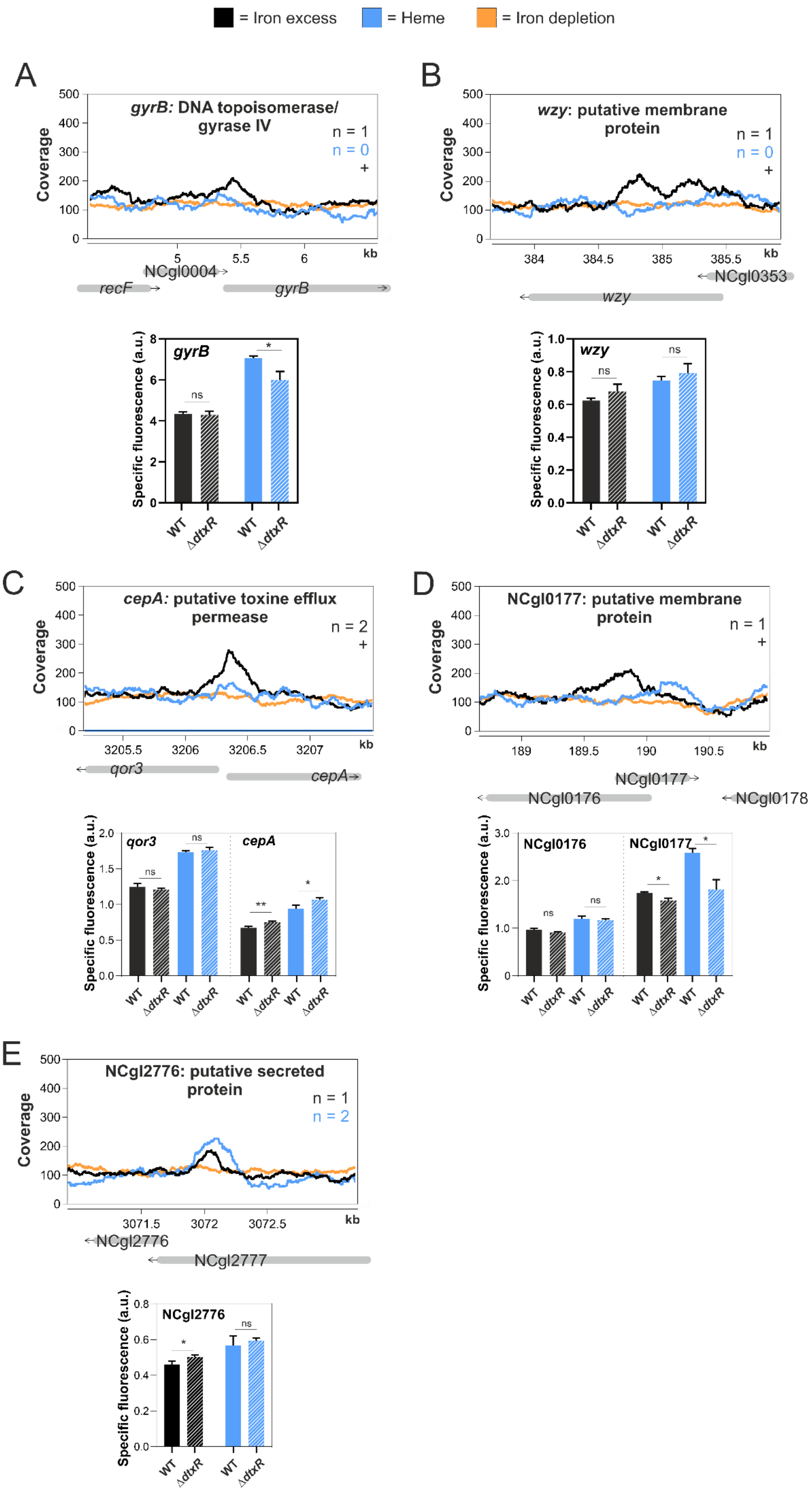

Figure S10: Binding peaks and reporter outputs of further selected novel DtxR targets. Representative ChAP-Seq binding peaks are shown for iron excess (black), heme (blue), and iron depletion (orange) conditions (see also Figure 3). The number of replicates with significant peaks is indicated as ‘n’ in the corresponding condition color. Bar plots display specific fluorescent reporter output after 2h of either *C. glutamicum* WT (filled bar) or a *dtxR* deletion strain Δ*dtxR* (striped bar) transformed with a reporter plasmid pJC1-P_x-*venus* (Table S1) coupling the expression of the respective promoter region of the respective target gene (x) to a *venus* fluorescent output. Statistical significance was confirmed by Student’s t-test (P value ≤ 0.05).

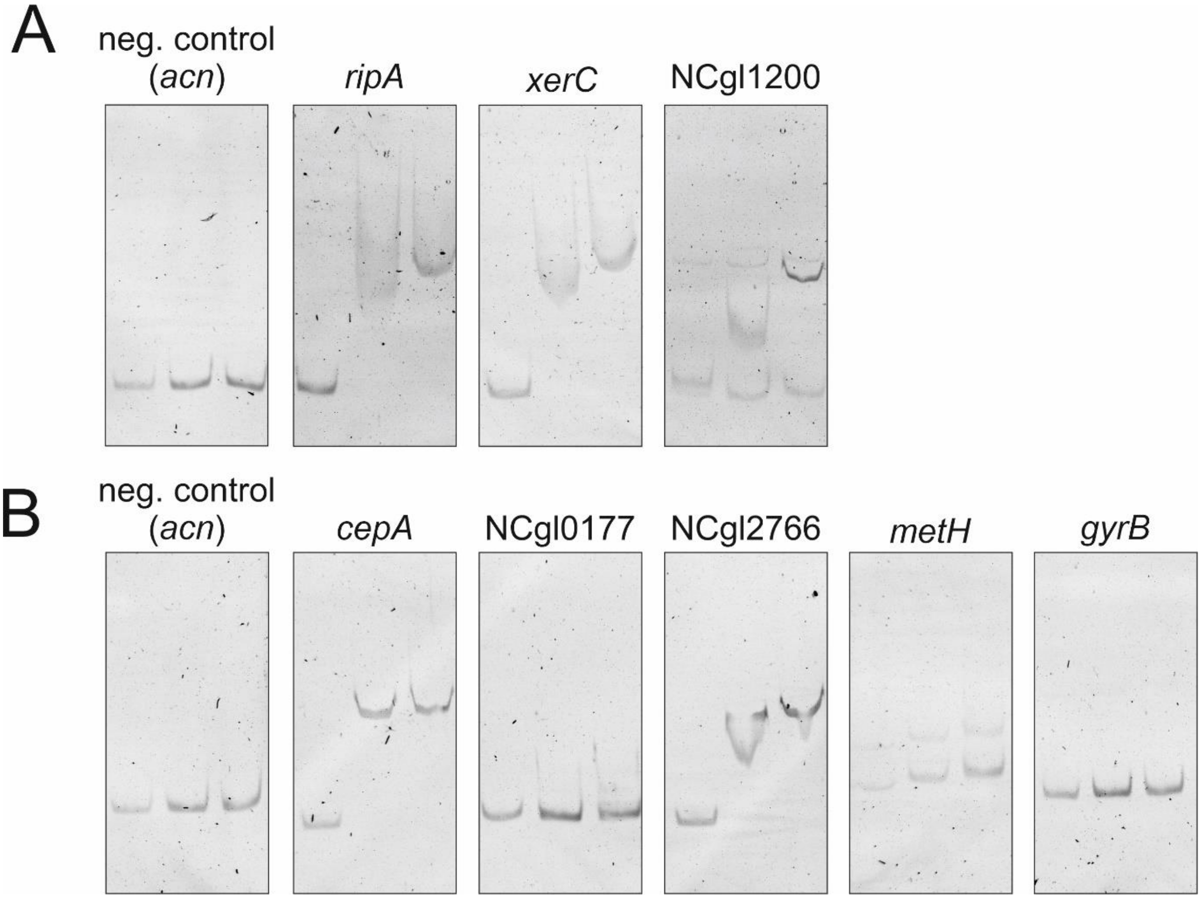

Figure S11: In vitro DtxR binding to selected weak targets identified by ChAP-Seq. Electron mobility shift assays were performed as described previously with minor adjustments (Wennerhold and Bott, 2006; Wennerhold et al., 2005). The expression plasmid pET24b-*dtxR*-C in *E. coli* BL21(DE3) was used for overproduction of DtxR of *Corynebacterium glutamicum* containing a His-Tag (HHHHLEHHHHHH) at the carboxyl terminus as previously described (Wennerhold and Bott, 2006). DtxR was purified using Ni-NTA agarose column material (Thermo Fisher Scientific, USA) according to manufacturer's instructions to the gravity flow protocol. Fractions containing DtxR were pooled and elution buffer was exchanged against TG buffer (30 mM Tris-HCl, 10% (v/v) glycerol, pH 7.5) using PD-10 desalting columns (Cytiva, USA). The purified DNA fragments of 121 bp size covering the region of identified ChAP-Seq peaks (61 bp peak extended 30 bp to each side; final concentration 31 nM) were incubated for 30 min at room temperature with (A) 0, 50 or 200-fold purified DtxR protein excess or (B) 0, 300 and 400-fold excess, respectively. The reaction buffer contained 50 mM Tris-HCl (pH 7.5), 5 mM $MgCl_2$, 40 mM KCl, 5% (v/v) glycerol, 1 mM dithiothreitol (DTT), and 150 µM $MnCl_2$. Afterwards,separation followed by a gel electrophoresis using a native polyacrylamide (10%) gel supplemented with 1 mM DTT and 150 µM $MnCl_2$. Electrophoresis was performed at 170 V using TB buffer (89 mM Tris, 89 mM boric acid) supplemented with 1 mM DTT and 150 µM $MnCl_2$ for 1.5 h. Gels were stained for 20 min with ethidium bromide. 121 bp promoter region of *acn* served as negative control. Protocol was adjusted according to Wennerhold and Bott (2006).

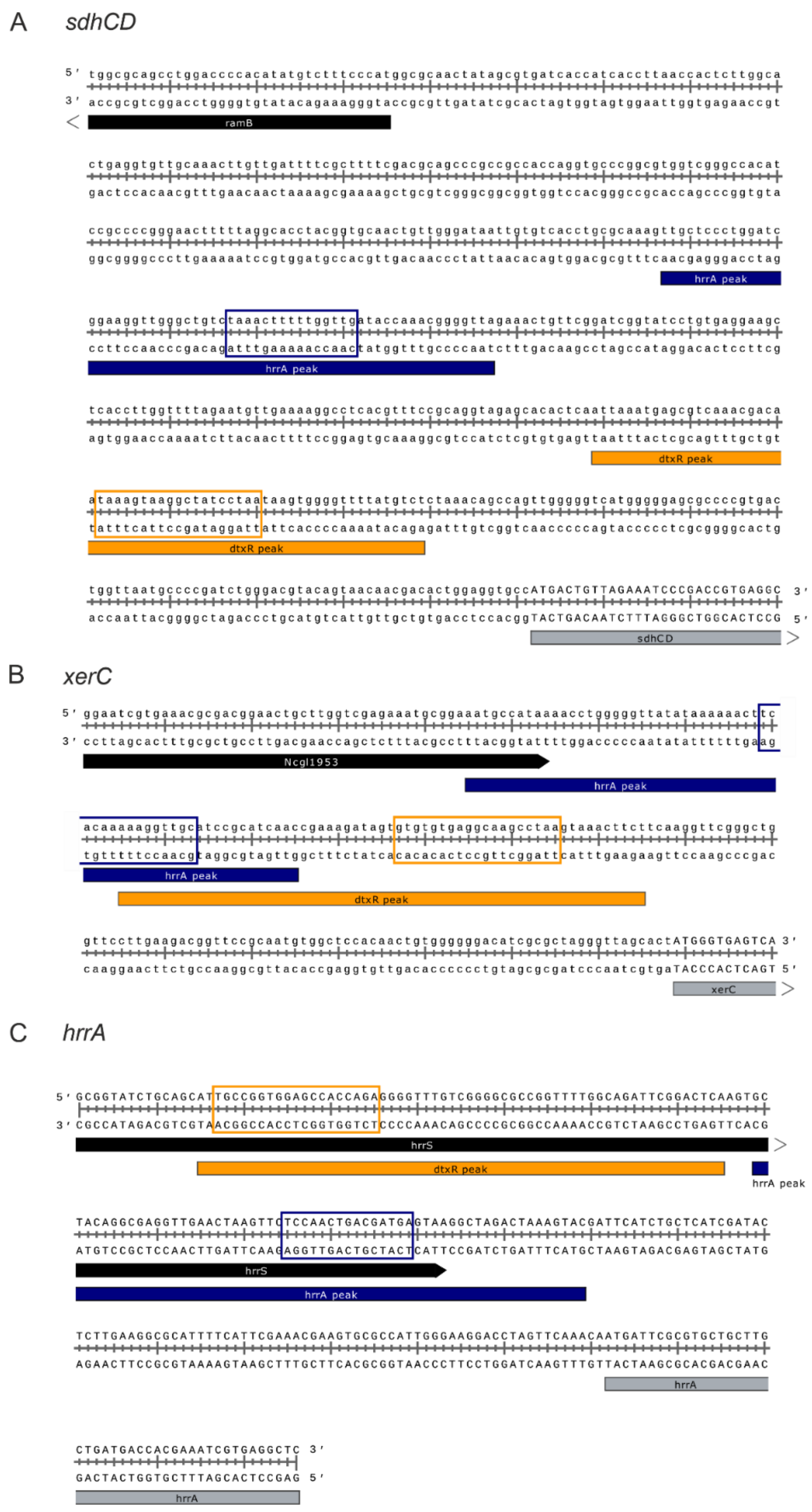

Figure S12: Location of DtxR and HrrA peaks in the promoter region of selected shared target genes. The sequences up- and downstream of the identified peaks corresponding to shared target genes of DtxR and HrrA including (A) *sdhCD*, (B) *xerC*, and (C) *hrrA* were extracted using SnapGene software (www.snapgene.com). The respective target gene is shown in grey. Further genes in that region are shown in black. The region of the DtxR peak is shown in orange and for HrrA in blue. The sequence corresponding to the actual binding motif as revealed by FIMO analysis (Grant et al., 2011) is highlighted in the respective color.

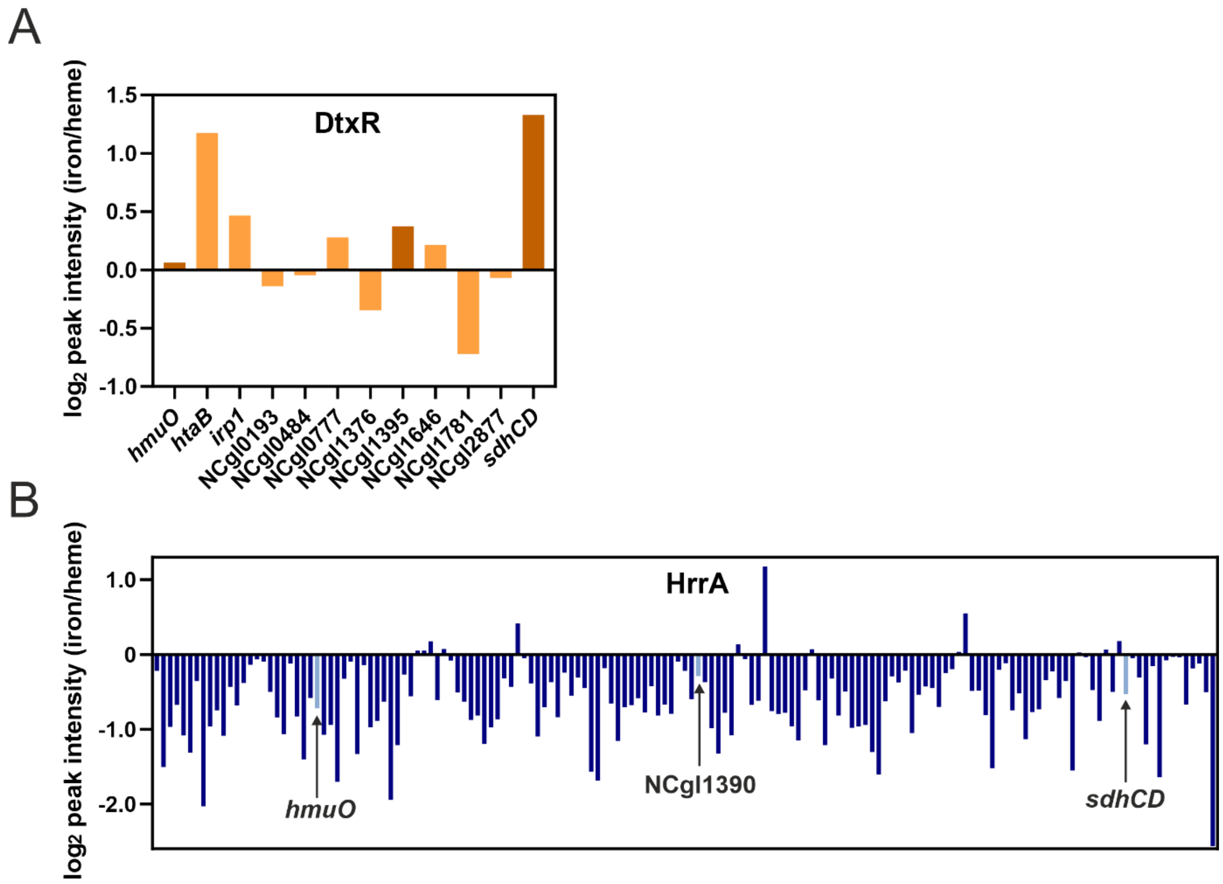

Figure S13: Ratios of iron and heme peak intensities. The ratios were calculated for targets with detectable signals in both conditions by dividing the mean peak intensity under iron conditions by that under heme conditions. Log2 transformation was applied, where values above 0 indicate stronger regulation under iron conditions, and values below 0 suggest stronger regulation under heme conditions. (A) Ratios for DtxR target binding, with targets shared with HrrA highlighted in dark orange. (B) Ratios for HrrA target binding, with shared targets with DtxR highlighted in light blue. The data demonstrate a trend for stronger binding under iron excess conditions for DtxR and under heme conditions for HrrA.

Table S1: Bacterial strains and plasmids used in this study.

Strain	Characteristics	Reference
Escherichia coli		
DH5α	F^- Φ80*lacZ*ΔM15 Δ(*lacZYA-argF*) U169 *recA1 endA1 hsdR17*(r_k^-, m_k^+) *phoA supE44 thi-1 gyrA96 relA1* λ^-; for general cloning purposes	Invitrogen
BL21(DE3)	*ompT hsdS*$_B$($r_B^- m_B^-$) *gal dcm* (DE3)	(Studier and Moffatt, 1986)
Corynebacterium glutamicum		
C. glutamicum ATCC13032	Biotin-auxotrophic wild type strain	(Kinoshita et al., 2004)
C. glutamicum ATCC13032::*dtxR*-C-linker-His	Derivative of ATCC 13032 encoding a C-terminally His-tagged version of DtxR (cg2103) with a flexible linker sequence in between (GGGGSGGGGS).	This work
C. glutamicum ATCC13032::*hrrA*-C-twinstrep	Derivative of ATCC 13032 encoding a C-terminally twinstrep-tagged version of HrrA (cg3247).	(Keppel et al., 2020)
Plasmids		
pK19-*mobsacB*	Contains negative (*sacB*) and positive (Kan^r) selection markers for genomic integration and deletion, MCS cut with EcoRI/BamHI	(Schäfer et al., 1994)
pK19-*mobsacB*-*dtxR*-C-linker-His	Derivative of pK19*mobsacB* for C-terminal integration of a His-Tag and a flexible linker in between; Kan^r	This work
pJC1-*venus*-term-BS	*E. coli-C. glutamicum* shuttle vector; derivative of pJC1 (Cremer et al., 1990), containing the terminator sequence of *Bacillus subtilis* behind venus; *oriV*$_{Ec}$ *oriV*$_{Cg}$; Kan^r	(Baumgart et al., 2013)
pJC1-P$_{NCgl1781}$-*venus*	Derivative of pJC1-*venus*-term-BS, containing *venus* under the control of the promoter P$_{NCgl1781}$	This work
pJC1-P$_{xerC}$-*venus*	Derivative of pJC1-*venus*-term-BS, containing *venus* under the control of the promoter P$_{xerC}$	This work
pJC1-P$_{qor3}$-*venus*	Derivative of pJC1-*venus*-term-BS, containing *venus* under the control of the promoter P$_{qor3}$	This work
pJC1-P$_{cepA}$-*venus*	Derivative of pJC1-*venus*-term-BS, containing *venus* under the control of the promoter P$_{cepA}$	This work
pJC1-P$_{NCgl2766}$-*venus*	Derivative of pJC1-*venus*-term-BS, containing *venus* under the control of the promoter P$_{NCgl2766}$	This work
pJC1-P$_{metH}$-*venus*	Derivative of pJC1-*venus*-term-BS, containing *venus* under the control of the promoter P$_{metH}$	This work

pJC1-P$_{NCgl1451}$-*venus*	Derivative of pJC1-*venus*-term-BS, containing *venus* under the control of the promoter P$_{NCgl1451}$	This work
pJC1-P$_{NCgl0176}$-*venus*	Derivative of pJC1-*venus*-term-BS, containing *venus* under the control of the promoter P$_{NCgl0176}$	This work
pJC1-P$_{NCgl0177}$-*venus*	Derivative of pJC1-*venus*-term-BS, containing *venus* under the control of the promoter P$_{NCgl0177}$	This work
pJC1-P$_{gyrB}$-*venus*	Derivative of pJC1-*venus*-term-BS, containing *venus* under the control of the promoter P$_{gyrB}$	This work
pJC1-P$_{wzy}$-*venus*	Derivative of pJC1-*venus*-term-BS, containing *venus* under the control of the promoter P$_{wzy}$	This work
pET24b-*dtxR*-C	pET24b derivative for overproduction of DtxR with a C-terminal decahistidine tag; the four additional histidines were attached to the *dtxR* fragment; Kan^r	(Wennerhold et al., 2005)

Table S2: Oligonucleotides used in this study provided by Eurofins Genomics (Germany).

Oligonucleotide	Sequence 5’ → 3’	Usage
A312-dtxR-qPCR-fw	TTGAGCAGTTCCAGGCACTC	qPCR primer for *dtxR*
A313-dtxR-qPCR-rv	TTTCTACGCGGACTGCATGT	
A156-*ddh*-qPCR-fw	CCGGAAAGCAAACCCACAAG	qPCR primer for housekeeping gene *ddh* (Frunzke et al., 2008)
A157-*ddh*-qPCR-rv	CTCGGAGTCGAAGGTTGCTT	
A280-leftflank-dtxR-C_fw	**CAGGTCGACTCTAGAGGATC**GACGGTCTTGTGCACGTCAGC	Left flank for tag integration to *dtxR*
A290-leftflank-dtxR-His-C-2-rv	TGATGATGATGTGAGCCGCCGCCGCCTGAGCCGCCGCCGCCGCCCTCAACCTTTTCTACGC	
A291-rightflank-dtxR-His-C-2-fw	GCGGCGGCTCACATCATCATCATCATCATCATCATCATCATTAACACACGATGAAGCTTCT	Right flank for tag integration to *dtxR*
A283-rightflank-dtxR-C-rv	**GTAAAACGACGGCCAGTGAATTG**CGATGTTTCCGTATGCGCCAGC	
A296-dtxR-Tag-C-seq-fw	CAGTCCGGCCCAACTGTCAG	Sequencing primers for tag integration
A297-dtxR-Tag-C-seq-rv	CCGGTTGCTACCTGCAGCAC	
A569_NCgl1781_fw	**AGCGACGCCGCAGGGGGATC**CTCCAATACAAGCGCTGCTACC	Reporter plasmid insert with promoter region of NCgl1781
A570_NCgl1781_rv	**ATGATATCTCCTTCTTAAAGTTCA**TGCAGGGGCTGGGGTATCAG	
A571_xerC_fw	**AGCGACGCCGCAGGGGGATC**GAGTATCGCTACCGAAGCCG	Reporter plasmid insert with promoter region of *xerC*
A572_xerC_rv	**ATGATATCTCCTTCTTAAAGTTCA**ACGCGCCACCTTCTTTTTTGACTC	
A573_qor3_fw	**AGCGACGCCGCAGGGGGATCCTA**TGTGCCTGCGCCACATTCTG	Reporter plasmid insert with promoter region of *qor3*
A574_qor3_rv	**ATGATATCTCCTTCTTAAAGTTCA**GGGATCAGGGGTATCCATTTGC	
A575_cepA_fw	**AGCGACGCCGCAGGGGGATC**GGCTACCAGCTGAGTTAGATTGATAAC	Reporter plasmid insert with promoter region of *cepA*
A576_cepA_rv	**ATGATATCTCCTTCTTAAAGTTCA**CCGTGCTTCGGGCGCGGT	
A577_NCgl2766_fw	**AGCGACGCCGCAGGGGGATC**CACATCCCCACGTTACCCCA	Reporter plasmid insert with promoter region of NCgl2766
A578_NCgl2766_rv	**ATGATATCTCCTTCTTAAAGTTCA**TTTGGTCGAAGAAGGAGTGGGC	
A587_metH_fw	**AGCGACGCCGCAGGGGGATCCTA**AGAGGTGGCTTTTACGTCGTCAAG	Reporter plasmid insert with promoter region of *metH*
A588_metH_rv	**ATGATATCTCCTTCTTAAAGTTCA**GGTGCCCATGGCGCCGTC	
A648_NCgl1451_fw	**AGCGACGCCGCAGGGGGATCCTA**CATCAGGGCGGGTGTCGTTG	Reporter plasmid insert with promoter region of NCgl1451
A649_NCgl1451_rv	**ATGATATCTCCTTCTTAAAGTTCA**TGTTGAGCCTTGCGGAGTGG	
A593_NCgl0176_fw	**AGCGACGCCGCAGGGGGATCCTA**TCCAAACTCATCCGCTGCAGTTT	

A594_NCgl0176_rv	ATGATATCTCCTTCTTAAAGTTCAACGGGCG GCGTCAGTTAAC	Reporter plasmid insert with promoter region of NCgl0176
A595_NCgl0177_fw	**AGCGACGCCGCAGGGGGATCCTA**GGCGAGTA CTTTGTCACGGG	Reporter plasmid insert with promoter region of NCgl0177
A596_NCgl0177_rv	ATGATATCTCCTTCTTAAAGTTCAATCGTGG AGTCTTCTAAATTTTCGAGT	
A652_gyrB_fw	**AGCGACGCCGCAGGGGGATCCTA**TGGGCCAC CAATCTGCGCAT	Reporter plasmid insert with promoter region of *gyrB*
A653_gyrB_rv	ATGATATCTCCTTCTTAAAGTTCAAGCGTCA TAATTGTGTTCAGTGTTTG	
A656_wzy_fw	**AGCGACGCCGCAGGGGGATCCTA**AAGCGGTC ACCAGTTCGAC	Reporter plasmid insert with promoter region of *wzy*
A657_wzy_rv	ATGATATCTCCTTCTTAAAGTTCAACTTCCG ACAGCTAAAGGCTCAC	
A625_RBSlinker_venus_fw	TGAACTTTAAGAAGGAGATATCATATGGTGA GCAAGGGCGAGGA	Venus insert for reporter plasmid
A626_venus_rv	**AAAACGACGGCCAGTACTAGTTTA**CTTGTAC AGCTCGTCCATGCC	
A796-metH-EMSA_fw	GCTGGTGAAGTAACTGAAGTAGACATTG	121 bp of *metH* covering ChAP-seq peak, for EMSAs
A797-metH-EMSA_rv	GTGGCGCTTTGCCTGTGTTG	
A798-xerC-EMSA_fw	CCGTCTTCAAGGAACCAG	121 bp of *xerC* covering ChAP-seq peak, for EMSAs
A799-xerC-EMSA_rv	AACCTGGGGGTTATATAAAAAACTTC	
A800-ripA-EMSA_fw	ATGTAACATCATTTCGAAGATATGAG	121 bp of *ripA* covering ChAP-seq peak, for EMSAs
A801-ripA-EMSA_rv	AACTGTTGACACACCGGAATG	
A802-NCgl1200-EMSA_fw	ATTGGCTGCCCTCGCATG	121 bp of NCgl1200 covering ChAP-seq peak, for EMSAs
A803-NCgl1200-EMSA_rv	CTTATCGATATATCTTTAACTGACCTAG	
A804-gyrB-EMSA_fw	GGACCTAAGCGTGTAAGATG	121 bp of *gyrB* covering ChAP-seq peak, for EMSAs
A805-gyrB-EMSA_rv	CAAGACCTTCAAGGATGGTG	
A806-wzy-EMSA_fw	AGCTCCGCCACCAGCAATTC	121 bp of *wzy* covering ChAP-seq peak, for EMSAs
A807-wzy-EMSA_rv	CTTCCTTTTCTTAGTAACCAGTTCTTTC	
A808-cepA-EMSA_fw	TTTGACGTGCTTTTTTAGCACG	121 bp of *cepA* covering ChAP-seq peak, for EMSAs
A809-cepA-EMSA_rv	CTTCATGTGGTAAATGACAATGCG	
A810-NCgl0177-EMSA_fw	GATTGGTGCTTAAGTAATGGACTG	121 bp of NCgl0177 covering ChAP-seq peak, for EMSAs
A811-NCgl0177-EMSA_rv	GAGTAGTAGCAGTGGGTATTTGC	
A812-NCgl2766-EMSA_fw	TAGTCGTATCAAAAAGTGCTCTG	121 bp of NCgl2766 covering ChAP-seq peak, for EMSAs
A813-NCgl2766-EMSA_rv	TTGCAATGTTTGTTAATAAATGGGTTC	
A814-hmuO-EMSA_fw	GATTCCTCAGAGAAATCCTCACG	121 bp of *hmuO* covering ChAP-seq peak, for EMSAs
A815-hmuO-EMSA_rv	AGTTTTAGGTAGGTGGTGGG	

bold = overlap to backbone, orange = flank overlap, EMSA = electron mobility shift assay

Table S5: Overlapping peaks for DtxR and HrrA.

gene number	gene	DtxR iron excess		DtxR heme		HrrA iron excess		HrrA heme	
		µ peak intensity	n	µ peak intensity	n	µ peak intensity	n	µ peak intensity	n
NCgl0005	*gyrB*	2.01	1	n.d.	0	2.80	2	n.d.	0
NCgl0177/ NCgl0176		2.03	1	n.d.	0	2.42	3	3.45	1
NCgl0352	*wzy*	1.94	1	n.d.	0	5.44	3	7.71	3
NCgl0353		n.d.	0	2.02	1	n.d.	0	3.38	1
NCgl0358/ NCgl0359	*ramB/ sdhCD*	7.93	3	3.16	3	50.47	3	72.92	3
NCgl1200		2.24	1	n.d.	0	n.d.	0	2.50	1
NCgl1395		3.31	3	2.56	3	3.07	3	3.75	3
NCgl1444		2.23	2	n.d.	0	9.48	3	12.28	3
NCgl1952	*xerC*	n.d.	0	6.54	3	43.06	3	254.09	3
NCgl2146	*hmuO*	16.51	3	15.80	3	3.01	3	4.96	3
NCgl2766		2.29	3	n.d.	0	3.33	2	n.d.	0
NCgl2834	*hrrA*	n.d.	0	3.19	2	2.99	3	6.29	3
NCgl2842	*uspA3*	n.d.	0	2.02	1	3.64	2	n.d.	0
NCgl2903/ NCgl2902	*cepA/ qor3*	2.53	2	n.d.	0	n.d.	0	3.11	3
NCgl2968		1.89	1	n.d.	0	4.71	3	6.75	3
NCgl2980		n.d.	0	2.00	1	2.25	2	n.d.	0

µ = mean value, n = number of replicates with a peak identified, n.d. = no peak detected, underlined = peak was annotated for HrrA for the gene into the other direction, i.e. binding probably not at same site.

References

Bailey, T.L., and Elkan, C. (1994). Fitting a mixture model by expectation maximization to discover motifs in biopolymers. Proc Int Conf Intell Syst Mol Biol, 2: 28-36.

Baumgart, M., Luder, K., Grover, S., Gätgens, C., Besra, G.S., and Frunzke, J. (2013). IpsA, a novel LacI-type regulator, is required for inositol-derived lipid formation in *Corynebacteria* and *Mycobacteria*. BMC Biol, 11: 122. doi: 10.1186/1741-7007-11-122.

Brune, I., Werner, H., Hüser, A.T., Kalinowski, J., Pühler, A., and Tauch, A. (2006). The DtxR protein acting as dual transcriptional regulator directs a global regulatory network involved in iron metabolism of *Corynebacterium glutamicum*. BMC Genomics, 7(1): 21. doi: 10.1186/1471-2164-7-21.

Cremer, J., Eggeling, L., and Sahm, H. (1990). Cloning the *dapA dapB* cluster of the lysine-secreting bacterium *Corynebacterium glutamicum*. Molecular and General Genetics MGG, 220(3): 478-480. doi: 10.1007/BF00391757.

Frunzke, J., Bramkamp, M., Schweitzer, J.E., and Bott, M. (2008). Population Heterogeneity in *Corynebacterium glutamicum* ATCC 13032 caused by prophage CGP3. J Bacteriol, 190(14): 5111-5119. doi: 10.1128/JB.00310-08.

Grant, C.E., Bailey, T.L., and Noble, W.S. (2011). FIMO: scanning for occurrences of a given motif. Bioinformatics, 27(7): 1017-1018. doi: 10.1093/bioinformatics/btr064 %J Bioinformatics.

Kensy, F., Zang, E., Faulhammer, C., Tan, R.-K., and Büchs, J. (2009). Validation of a high-throughput fermentation system based on online monitoring of biomass and fluorescence in continuously shaken microtiter plates. Microb Cell Factories, 8(1): 31. doi: 10.1186/1475-2859-8-31.

Keppel, M., Hünnefeld, M., Filipchyk, A., Viets, U., Davoudi, C.F., Krüger, A., Mack, C., Pfeifer, E., Polen, T., Baumgart, M.*, et al.* (2020). HrrSA orchestrates a systemic response to heme and determines prioritization of terminal cytochrome oxidase expression. Nucleic Acids Research, 48(12): 6547–6562. doi: 10.1093/nar/gkaa415.

Kinoshita, S., Udaka, S., and Shimono, M. (2004). Studies on the amino acid fermentation. Part 1. Production of L-glutamic acid by various microorganisms. The Journal of General and Applied Microbiology, 50(6): 331-343.

Livak, K.J., and Schmittgen, T.D. (2001). Analysis of relative gene expression data using real-time quantitative PCR and the $2^{-\Delta\Delta Ct}$ Method. Methods, 25(4): 402-408. doi: 10.1006/meth.2001.1262.

Madeira, F., Madhusoodanan, N., Lee, J., Eusebi, A., Niewielska, A., Tivey, A.R.N., Lopez, R., and Butcher, S. (2024). The EMBL-EBI Job Dispatcher sequence analysis tools framework in 2024. Nucleic acids research, 52(W1): W521-W525. doi: 10.1093/nar/gkae241.

Schäfer, A., Tauch, A., Jager, W., Kalinowski, J., Thierbach, G., and Pühler, A. (1994). Small mobilizable multi-purpose cloning vectors derived from the *Escherichia coli plasmids* pK18 and pK19: selection of defined deletions in the chromosome of *Corynebacterium glutamicum*. Gene, 145(1): 69-73.

Studier, F.W., and Moffatt, B.A. (1986). Use of bacteriophage T7 RNA polymerase to direct selective high-level expression of cloned genes. J Mol Biol, 189(1): 113-130. doi: 10.1016/0022-2836(86)90385-2.

Wennerhold, J., and Bott, M. (2006). The DtxR regulon of *Corynebacterium glutamicum*. Journal of Bacteriology, 188(8): 2907-2918. doi: 10.1128/jb.188.8.2907-2918.2006.

Wennerhold, J., Krug, A., and Bott, M. (2005). The AraC-type Regulator RipA Represses Aconitase and Other Iron Proteins from *Corynebacterium* under Iron Limitation and Is Itself Repressed by DtxR. Journal of Biological Chemistry, 280(49): 40500-40508. doi: https://doi.org/10.1074/jbc.M508693200.
